# TRIM6 ablation reverses ICB resistance in MSS gastric cancer by unleashing cGAS-STING-dependent antitumor immunity

**DOI:** 10.1186/s13046-025-03513-5

**Published:** 2025-08-15

**Authors:** Yinan Niu, Chen Ding, Quansheng Wang, Jingyi Yin, Lingmeng Li, Wenshuai Liu, Xuefei Wang, Liyu Huang

**Affiliations:** 1 Key Laboratory of Systems Biomedicine (Ministry of Education) and Collaborative Innovation Center of Systems Biomedicine, Shanghai Center for Systems Biomedicine, Shanghai Jiao Tong University, 800 Dong Chuan Road, Shanghai, 200240 China; 2 Department of Gastrointestinal Surgery, Zhongshan Hospital, Fudan University, 180 Fenglin Road, Shanghai, 200032 China; 3 Retroperitoneal Sarcoma Center, Zhongshan Hospital, Fudan University, 180 Fenglin Road, Shanghai, 200032 China

**Keywords:** TRIM6, cGAS, MSS, Immunotherapy, Ubiquitination, Gastric cancer

## Abstract

**Background:**

Gastric cancers are classified into four molecular subtypes according to The Cancer Genome Atlas (TCGA) classification: Epstein-Barr virus-positive (EBV-positive), microsatellite instability-high (MSI-H), chromosomal instability (CIN), and genomically stable (GS). Unlike MSI-H gastric cancer, GS and CIN subtypes exhibit immunologically inert microenvironments and demonstrate minimal response to immune checkpoint blockade (ICB), necessitating novel strategies to overcome immunotherapy resistance.

**Methods:**

Through weighted gene co-expression network analysis (WGCNA), we identified the E3 ubiquitin ligase TRIM6 as inversely associated with MSI-H status. *TRIM6*-knockout murine models and subcutaneous tumors were subjected to flow cytometry, RNA sequencing, immunoblotting, and ubiquitination assays to characterize tumor-infiltrating lymphocytes (TILs), pathway activation, and TRIM6-mediated regulation of the cGAS-STING axis.

**Results:**

Hypermethylation-mediated *TRIM6* downregulation distinguished MSI-H from microsatellite stable (MSS) gastric cancers. Clinically, *TRIM6* expression inversely correlated with cytotoxic T lymphocyte (CTL) infiltration and anti-PD-1/PD-L1 therapeutic efficacy. Mechanistically, TRIM6 catalyzed K27-linked polyubiquitination of cGAS, triggering its proteasomal degradation and consequent suppression of the cGAS-STING pathway. *TRIM6* ablation enhanced CD8^+^ T lymphocytes infiltration via cGAS-mediated innate immune response and synergized with anti-PD-L1 therapy in MSS gastric tumors.

**Conclusions:**

Our results elucidate TRIM6-mediated suppression of antitumor immunity as a novel mechanism underlying ICB resistance in MSS gastric cancer, positioning TRIM6 as both a predictive biomarker and therapeutic target for immunologically cold subtypes.

**Supplementary Information:**

The online version contains supplementary material available at 10.1186/s13046-025-03513-5.

## Background

Gastric cancer (GC) ranks as the fifth most frequently diagnosed malignancy and the fourth leading cause of cancer-related mortality worldwide [1]. The Cancer Genome Atlas (TCGA) classification system divides gastric adenocarcinoma into four molecular subtypes: Epstein-Barr virus-positive (EBV-positive, 9%), microsatellite instability-high (MSI-H, 22%), chromosomal instability (CIN, 20%) and genomically stable (GS, 50%) [2]. The Asian Cancer Research Group (ACRG) classification system categorizes gastric cancer into four molecular subtypes: MSI (23%), microsatellite stable with epithelial-to-mesenchymal transition (MSS/EMT, 15%), MSS/TP53^+^ (26%), and MSS/TP53^−^ (36%) [3]. Among these subtypes, EBV-positive and MSI-H gastric cancers demonstrate remarkable sensitivity to immunotherapy, while GS and CIN types show minimal response to immune checkpoint blockade (ICB) [2]. Clinical trials have consistently shown high efficacy of pembrolizumab in these immunogenic subtypes, with EBV-positive GC patients exhibiting a 100% overall response rate (ORR) and MSI-H GC showing an 85.7% ORR [4–8]. In stark contrast, GS and CIN subtypes exhibit limited or no response to anti-PD-1/PD-L1 therapy [7], reflecting their immunologically ‘cold’ tumor microenvironments.

Recent research efforts to overcome immunotherapy resistance in cold tumors have uncovered several promising strategies. In gastric cancer, inhibition of the IL-8/CXCR2 pathway reduces PD-L1 expression and enhances anti-PD-1 efficacy [9]. Similarly, Siglec-10 blockade reinvigorates antitumor immunity and synergizes with anti-PD-1 therapy [10]. Targeting VISTA reprograms tumor-associated macrophages to promote T-cell-mediated immunity and improve PD-1 inhibitor response [11]. For peritoneal metastases, disrupting the C3-C3AR1 axis breaks the CAF-macrophage immunosuppressive niche, boosting ICB efficacy [12].

Despite these advances, the molecular basis for immunotherapy resistance in MSS-type gastric carcinomas remains poorly understood. Emerging evidence highlights E3 ubiquitin ligases as critical regulators of immune regulation. Key players like FBXO38, Cullin3-SPOP, and TRAF6 directly modulate immune checkpoint proteins (PD-1, PD-L1, and CTLA-4) to shape antitumor immunity [13–15]. Our study employed weighted gene co-expression network analysis (WGCNA) of TCGA-STAD data, identifying TRIM6 as a RING-type E3 ligase inversely correlated with MSI-H status. TRIM6 shows hypermethylation-associated downregulation in MSI-H versus MSS tumors and negatively correlates with cytotoxic T lymphocyte infiltration and pembrolizumab response. Mechanistically, TRIM6 promotes proteasomal degradation of cGAS, suppressing the cGAS-STING pathway. Genetic ablation of *TRIM6* activates innate immunity and sensitizes cold gastric tumors to anti-PD-L1 therapy. These findings establish TRIM6 as both a novel regulator of immunotherapy response and a promising target for cold gastric cancers.

## Methods and materials

### Primary gastric cancer tissue samples

Gastric cancer tissue samples were provided by Zhongshan Hospital. Different subtypes of gastric cancer were confirmed by pathological analysis. Patients’ written informed consent was obtained for sample use, and this study was approved by the Ethics Committee of Fudan University (Approval Number: B2021-449R). For protein extraction, tissue samples stored at -80 °C were ground into fine powder in liquid nitrogen, and subsequently added RIPA lysis buffer for protein extract. DNA extraction was performed using the TIANamp Genomic DNA Kit (TIANGEN BIOTECH, #DP304-02).

### Cell lines

Human gastric cancer cell line SNU668 was obtained from Cobioer Biosciences (Nanjing, China); MKN28 was provided from the Gastric Cancer Center of Fudan University; Human embryonic kidney 293T (HEK293T) cell line was obtained from Dr. Qisheng He (Suzhou Medical College of Soochow University, Soochow University). Mouse gastric cancer cell line MTC was obtained from Dr. Wenshuai Liu (Zhongshan Hospital, Fudan University). Mouse gastric cancer cell line MFC was obtained from Cobioer Biosciences. Mouse melanoma B16F10 cell line was obtained from Professor Yujia Cai (Shanghai Center for Systems Biomedicine, Shanghai Jiao Tong University). All cells were cultured in DMEM (Gibco) containing 10% FBS (Gibco) and 1% penicillin-streptomycin, under 37 °C and 5% CO₂ conditions. All the cell lines were routinely tested negative for mycoplasma contamination and cultured according to the instructions of the ATCC.

### Mice and animal experiments

C57BL/6J mice harboring *Trp53*^fl/fl^ and *Trim6*^fl/fl^ alleles with *Villin*-Cre mice were obtained from GemPharmatech Co., Ltd. (Nanjing, China). The novel transgenic gastric cancer model was established by Shouzheng Pharma Biotechnology Co., Ltd. (Wuhan, China). Briefly, the transgenic mouse model of gastric cancer was generated by orthotopic co-delivery of plasmids (pT3-*Myc* and pCAG-SB100) into the gastric mucosa of 6-week-old *Trp53*^fl/fl^; *Villin*-Cre and *Trp53*^fl/fl^; *Trim6*^fl/fl^; *Villin*-Cre mice, with subsequent tissue analysis conducted following a critical 3-week induction period required for tumorigenesis. 6-week-old female C57BL/6J and BALB/c nude mice were purchased from GemPharmatech Co., Ltd., while 615 mice were obtained from the Blood Institute of the Chinese Academy of Medical Sciences (Tianjin, China).

For syngeneic mice subcutaneous tumor models experiments, 4 × 10^6^ MTC, or 1 × 10^5^ B16F10 cells in 200 µL phosphate-buffered saline (PBS) were injected subcutaneously into 6-week-old C57BL/6J and BALB/c nude mice. 1 × 10^6^ MFC cells were injected subcutaneously into 6-week-old 615 and BALB/c nude mice. In the MTC tumor model, anti-PD-L1 (200 µg/mouse) was initiated at 4 weeks post-implantation and maintained every two weeks until experimental endpoint was reached. In the B16F10 tumor model, anti-PD-L1 (200 µg/mouse) treatment was administered twice weekly, beginning 7 days after tumor cell inoculation. In the MFC tumor model, the same anti-PD-L1 antibody was administered via intraperitoneal injection on Days 6, 9, and 12 post-tumor inoculation, with isotype antibodies serving as controls. Tumor volume was measured every 2–3 days using a vernier caliper and calculated with the formula: (tumor length × tumor width²)/2. Mice were euthanized upon reaching the 2000 mm³ tumor limit. All mice were maintained in the specific pathogen-free animal facility of Shanghai Jiao Tong University Laboratory Animal Center. All experiments were done according to the guidelines of the Institutional Animal Care and Use Committee at the Shanghai Jiao Tong University (Approval Number: A2024358).

### Plasmid

Human *TRIM6* (full-length) and its mutants (*TRIM6* C43A, ΔRING, ΔBbox, ΔCC, ΔPRY-SPRY), *cGAS* (full-length, RD domain, NTase domain, and C-terminal domain), *STING*, *IRF3* and *TBK1* were cloned into expression vectors including pcDNA3.1-Flag, pcDNA3.1-HA, and pcDNA3.1-Myc. Mouse *Trim6* was cloned into pCDH-GFP. For GST-pull down plasmids, *TRIM6* was cloned into the pGEX-6T-1 vector and *cGAS* into the pET28a plasmid. For CRISPR-Cas9 plasmids, single guide RNAs targeting human and mouse *TRIM6* and *cGAS* were designed by CRISPOR [16] and cloned into pLentiCRISPRv2. The shRNA sequences for *TRIM6* were cloned into pLKO.1 vector. The primer sequences are listed in Supplementary Table S1.

### Antibodies

The anti-human cGAS (#83623), anti-mouse cGAS (#31659; RRID: AB_2799008), anti-p-STING (Ser365, #62912; RRID: AB_2799635), anti-p-IRF3 (Ser 396, #29047; RRID: AB_2773013), and anti-p-TBK1 (Ser172, #5483; RRID: AB_10693472) antibodies were purchased from Cell Signaling Technology. Anti-p-STING (Ser366, #HA723137) antibody was purchased from HUABIO. Anti-TRIM6 antibodies (#SAB1306751, #orb681955, and #abs104444) were purchased from Sigma-Aldrich, Biorbyt, and Absin. Anti-TBK1 (#T55145) and anti-IRF3 (#T55779; RRID: AB_2936988) antibodies were obtained from Abmart. Anti-STING antibodies (#F0361 and #ET1705-68) were obtained from Selleck and HUABIO. Anti-Flag (#66008-4-Ig; RRID: AB_2918475), anti-HA (#51064-2-AP; RRID: AB_11042321), anti-Myc (#60003-2-Ig; RRID: AB_2734122), and anti-GAPDH (#60004-1-Ig; RRID: AB_2107436) antibodies were purchased from Proteintech. Rabbit IgG was purchased from Beyotime (#A7016; RRID: AB_2905533). Flow antibodies were purchased from BioLegend as follows: anti-mouse CD45 (30-F11, #103132; RRID: AB_893344); anti-mouse CD4 (GK1.5, #100422; RRID: AB_312707, GK1.5, #100405; RRID: AB_312690); anti-mouse CD8a (53-6.7, #100722; RRID: AB_312761, QA17A07, #155004; RRID: AB_2750211); anti-Granzyme B Recombinant (QA16A02, #372208; RRID: AB_2687032, QA18A28, #396413; RRID: AB_2810602); anti-mouse IFN-γ (XMG1.2, #505830; RRID: AB_2563105). Immunofluorescence secondary antibodies were purchased from Jackson ImmunoResearch as follows: Alexa Fluor 488-conjugated goat anti-mouse IgG (#115-545-003; RRID: AB_2338840) and Alexa Fluor 594-conjugated goat anti-rabbit IgG (#111-585-003; RRID: AB_2338059). For murine in vivo therapeutic administration, *InVivo*MAb anti-mouse PD-L1 (10 F.9G2™, #BE0101; RRID: AB_10949073), *InVivo*MAb rat IgG2b isotype control (LTF-2, #BE0090; RRID: AB_1107780), *InVivo*MAb anti-mouse CD8β (53-5.8, #BE0223; RRID: AB_2687706), and *InVivo*MAb rat IgG1 isotype control (HRPN, #BE0088; RRID: AB_1107775) were purchased from BioXcell.

### Generation of CRISPR knockout cell lines

pLentiCRISPRv2 vectors containing single guide RNAs targeting human and mouse *TRIM6* and *cGAS* were transiently transfected into MKN28, SNU668, MTC, and B16F10 cells with lipofectamine 2000 (Thermo Fisher Scientific, #11668019) according to the manufacturer’s recommendations, and cells were selected under puromycin treatment at a predetermined optimal concentration. Knockout (KO) cell lines were validated through genomic PCR amplification followed by Sanger sequencing, with confirmed clones subsequently employed in downstream experiments.

### WGCNA analysis and MSI MANTIS scores

The WGCNA R package was used to construct the gene co-expression networks of TCGA-STAD dataset (374 tumors) focusing on more than 356 E3 ligases, including most members of the large E3 ligase family. First, a similarity matrix was generated from gene correlation coefficients and transformed into an adjacency matrix using a soft threshold power (β = 6). Next, a topological overlap matrix (TOM) was calculated to reduce noise and spurious connections. Hierarchical clustering with dynamic tree cutting (minimum module size = 30 genes) was then applied to TOM-based dissimilarity to identify co-expression modules. Highly correlated modules (*r* > 0.75) were further merged using average-linkage hierarchical clustering with a height cutoff of 0.25. Finally, biologically relevant modules were determined through module-trait association analysis using MSI MANTIS scores as the key phenotypic trait.

MANTIS is the next-generation sequencing (NGS)-based algorithm developed for detecting MSI. As a key clinical indicator in our WGCNA analysis, the MANTIS score provides a quantitative measure of MSI status, with higher values indicating a greater likelihood of instability [17]. These scores have been validated across multiple tumor types [18] and were precomputed by TCGA, with public availability through resources such as cBioPortal [19].

### RT-qPCR and RNA sequencing analysis

Total RNA was isolated with RNA isolater Total RNA Extraction Reagent (Vazyme, #R401-01), reverse-transcribed using the PerfectStart Uni RT-qPCR Kit (Transgene, #AUQ-01-V2), and analyzed by RT-qPCR on a LightCycler 480 system (Roche). Relative mRNA levels were normalized to GAPDH. Primer sequences have been deposited in Supplementary Table S2.

For RNA sequencing (RNA-seq) analysis, total RNA was extracted from wild-type (WT) and *Trim6* KO MTC tumors subcutaneously transplanted in C57BL/6J mice (*n* = 4 per group). Sequencing libraries were prepared using the VAHTS Universal V10 RNA-seq Library Prep Kit and sequenced on the DNBSEQ-T7 platform (MGI Tech) by OE Biotech Co., Ltd. (Shanghai, China), yielding approximately 40–48 million 150-bp paired-end reads per sample. Raw reads were quality-assessed with FastQC, filtered by Q30 > 90%, and adapter-trimmed using fastp to obtain clean reads. The clean reads were then aligned to the NCBI GRCm39 reference genome using HISAT2. Gene expression levels were quantified as FPKM, and raw counts were obtained with HTSeq-count. PCA analysis was performed based on normalized counts. Differential expression analysis thresholds were FDR-adjusted p-value (q) < 0.05 and |log2(fold change)| >1 using DESeq2 (for biological replicates). Gene Ontology (GO) enrichment analysis of differentially expressed genes was based on the hypergeometric distribution. Gene set enrichment analysis (GSEA) [20] used significance thresholds of nominal *p* < 0.05 and FDR < 0.25. All software parameters were documented for reproducibility.

### Immunoblotting analysis

For Immunoblotting analysis, cells were lysed in lysis buffer (50 mM Tris pH 7.4, 150 mM NaCl, 1% Triton X-100, 1% sodium deoxycholate, and 0.1% SDS). Lysates were centrifuged at 12,000 × g for 5 min at 4 °C, and supernatant protein concentrations were quantified using the Pierce BCA Protein Assay Kit (Thermo Fisher Scientific, #23225). The protein samples were resolved by SDS-PAGE, transferred onto PVDF membranes, and immunoblotted with indicated antibodies.

### Immunoprecipitation and IP-MS

For immunoprecipitation (IP) analysis, ANTI-FLAG M2 Affinity Gel (Sigma-Aldrich, #A2220), or Protein-A Sepharose beads (Beyotime, #P2193M) accompanied with selected antibodies were added into supernatant and incubated overnight at 4 °C. Beads were washed four times with lysis buffer, and bound proteins were eluted by boiling in SDS sample buffer or processed for downstream applications.

For Immunoprecipitation-Mass Spectrometry (IP-MS) analysis, MKN28 cells transiently expressing empty vector (EV), Flag-TRIM6, or Flag-TRIM6-ΔPRY-SPRY were lysed and subjected to anti-Flag immunoprecipitation as described above. Immunoprecipitated proteins were resolved by SDS-PAGE and visualized with Coomassie Brilliant Blue staining. Gel slices containing protein bands were excised, trypsin-digested, and analyzed by liquid chromatography-tandem mass spectrometry (LC-MS/MS) on an Easy nLC1200/Q Exactive plus (Thermo Fisher Scientific).

### GST pull-down

Recombinant His-cGAS, GST-TRIM6, and truncation mutants were expressed in *Escherichia coli Rosetta* (DE3) and purified using affinity chromatography. For pull-down experiments, BeyoGold™ GST-tag Purification Resin (Beyotime, #P2250) was equilibrated with protein binding buffer (50 mM Tris pH 8.0, 200 mM NaCl, 1 mM EDTA, 1% NP-40, 1 mM DTT, and 1 mM MgCl_2_). Purified proteins were incubated with the resin overnight at 4 °C with gentle agitation. Beads were washed four times with binding buffer, and bound proteins were eluted with elution buffer (50 mM Tris pH 8.0, 400 mM NaCl, 50 mM reduced glutathione, 1 mM EDTA, and 1 mM DTT). Eluates were resolved by SDS-PAGE and analyzed by immunoblotting with anti-His or anti-cGAS antibodies.

### Luciferase reporter assay

HEK293T cells plated in 12-well plates were transiently co-transfected with indicated plasmids, including pGL3-IFNβ, Flag-cGAS, HA-STING, Myc-TRIM6. At 48 h post-transfection, cells were lysed using Passive Lysis Buffer (YEASEN, #11402ES60). Lysates were centrifuged at 12,000 × g for 5 min at 4 °C, and 20 µL of supernatant was added into reaction system. Luminescence signals were quantified using the Synergy 2 multimode microplate reader (BioTek).

### Ubiquitination assay

Ubiquitination of cGAS was analyzed by denaturing immunoprecipitation (d-IP) as previously reported [21]. Briefly, cells were lysed in SDS-lysing buffer (62.5 mM Tris-HCl pH 6.8, 2% SDS, 10% glycerol, 1.5% β-mercaptoethanol), boiled for 10 min and then diluted 10-fold in native lysis buffer (50 mM Tris-HCl pH 7.4, 0.5% Triton, 200 mM NaCl, 10% glycerol). After centrifugation at 12,000 × g for 5 min, supernatants were incubated with ANTI-FLAG M2 Affinity Gel (Sigma-Aldrich, #A2220) overnight at 4 °C with rotation. Beads were washed four times with native lysis buffer. The immunocomplexes were resolved by SDS-PAGE and immunoblotted with antibodies as indicated.

### Quantification of cGAMP levels

Intracellular cGAMP levels in MKN28 and MTC cells were measured by cGAMP Elisa Kit (Cayman, #501700). Cells were transfected with HT-DNA (2 µg/mL) and harvested by Mammalian Protein Extraction Reagent (Thermo Fisher Scientific, #78503) 12 h after transfection. 50 µL supernatant was used for cGAMP ELISA. The experiment was performed according to the manufacturer’s protocol.

### Cycloheximide chase assay

Cells seeded in 12-well plates were treated with 40 µg/mL cycloheximide (CHX; MedChemExpress, #HY-12320) for the specified time. Cells were lysed in RIPA Lysis Buffer (Beyotime, #P0013B) and used for subsequent analysis.

### Flow cytometry

Tumor tissues were dissociated into small fragments and enzymatically digested using a solution containing 1 mg/mL Collagenase IV (Sigma-Aldrich, #C5138), 0.1 mg/mL hyaluronidase (Sigma-Aldrich, #H3506-100MG), and 125 U/mL DNase I (Sigma-Aldrich, #D5025-15KU) in RPMI-1640 medium supplemented with 10% fetal bovine serum (FBS). Digestion was carried out at 37 °C for 1–1.5 h with periodic agitation. The resulting cell suspension was sequentially filtered through a 70-µm cell strainer and centrifuged at 500 × g for 5 min at 4 °C. After removal of the supernatant, red blood cells were lysed through incubation with Red Blood Cell Lysis Buffer (Sangon Biotech, #B541001) for 2 min on ice, followed by two washes with ice-cold PBS. Cell density was normalized to 1 × 10^7^ cells/mL in 100 µL PBS, and viability assessment was performed using the Zombie Aqua™ Fixable Viability Kit according to the manufacturer’s protocol. Surface markers were stained for 1 h at 4 °C in the dark. Cells were then washed with FACS buffer (1.5% BSA in PBS) and fixed with eBioscience™ IC Fixation Buffer (from the eBioscience™ Foxp3/Transcription Factor Staining Buffer Set [Thermo Fisher Scientific, #00-5523-00]) for 30 min on ice. For intracellular cytokine detection, cells were permeabilized with 1× Permeabilization Buffer (from the same kit) and stained with anti-IFNγ and anti-GZMB antibodies at 4 °C for 1 h. The cells were then washed twice with FACS buffer. Finally, cells were resuspended in FACS buffer and analyzed using a BD LSRFortessa™ flow cytometer equipped with FACSDiva™ software (BD Biosciences).

### Immunofluorescence and immunohistochemistry

For Immunofluorescence (IF) analysis, cells grown on glass coverslips were fixed with freshly prepared 4% paraformaldehyde (PFA) for 15–30 min at 4 °C, followed by three PBS washes. Specimens were permeabilized with 0.2% Triton X-100 in PBS/1% BSA for 8 min and blocked with 5% BSA/PBS for 1 h at room temperature. Primary antibodies were incubated overnight at 4 °C, followed by washing four times with PBS containing 1% BSA. Secondary antibodies were incubated at a dilution of 1:500 in PBS containing 1% BSA for 1 h at room temperature. After three final PBS washes, coverslips were mounted using DAPI-containing antifade medium (Beyotime, #P0131) and imaged by Nikon A1Si Laser Scanning Confocal Microscope.

For Immunohistochemistry (IHC) analysis, tissues were fixed in formalin, embedded in paraffin, and sectioned. Deparaffinized sections underwent rehydration and antigen retrieval in citrate buffer (pH 6.0). After PBS rinses (pH 7.4), endogenous peroxidase activity was quenched with 3% hydrogen peroxide (25 min at room temperature in the dark). Sections were blocked with 3% BSA, sequentially incubated with primary/secondary antibodies, and visualized using DAB with Harris hematoxylin counterstaining. Following dehydration and clearing, sections were mounted for microscopic analysis.

### Bisulfite genomic sequence polymerase chain reaction (BSP)

DNA of tumor samples was extracted using TIANamp Genomic DNA Kit (TIANGEN, #DP304). Bisulfite conversion of DNA was performed using EpiJET Bisulfite Conversion Kit (Thermo Fisher Scientific, #K1461). 2 µL converted DNA was added to the PCR mixture. The PCR products were purified, cloned into the pMD19-T vector (TaKaRa, #6013), and subjected to sequencing analysis. The primer sequences are listed in Supplementary Table S2.

### Statistical analysis

Data are expressed as mean ± SD. In vitro and in vivo experiments were analyzed using a two-tailed Student’s *t*-test, while multiple group comparisons were analyzed using one-way ANOVA. All tests were two-sided, with statistical significance defined as *p < 0.05, **p < 0.01, and ***p < 0.001. Comparisons of survival curves were performed with the log-rank test.

### Data Availability

The results displayed here are partially based on The Cancer Genome Atlas (TCGA) data (https://portal.gdc.cancer.gov/), Asian Cancer Research Group (ACRG) database (GSE62254) [3]. The association between *TRIM6* and *MLH1* expression in gastric tumors was analyzed using TIMER2 [22] (http://timer.cistrome.org/). The association between *TRIM6* expression and methylation and its relationship with activated CD8^+^ T cell, central memory CD8^+^ T cell, effector memory CD8^+^ T cell and cytotoxic T lymphocytes (CTLs) in TCGA-STAD was analyzed using TISIB [23] (http://cis.hku.hk/TISIDB/index.php) and TIDE [24] (http://tide.dfci.harvard.edu). Gene expression profiles were compared between responders and non-responders in the GC_Immunotherapy_Combined dataset (PRJEB25780 and Zhongshan cohort [7, 25]) using the limma-trend method, with cohort incorporated as a covariate to adjust for batch effects. The publicly available gene expression and clinical data of gastric cancer and melanoma patients used in this study are available in the European Nucleotide Archive (ENA) with accession number PRJEB25780 and in the Gene Expression Omnibus (GEO) with accession number GSE91061 [26]. Kaplan-Meier Plotter (https://kmplot.com/analysis/) were used for overall survival curves of patients with gastric cancer.

The raw RNA-seq data reported in this paper have been deposited in the Genome Sequence Archive [27] in National Genomics Data Center [28], China National Center for Bioinformation/Beijing Institute of Genomics, Chinese Academy of Sciences (GSA: CRA025505) that are publicly accessible at https://ngdc.cncb.ac.cn/gsa.

## Results

### TRIM6 expression is inversely correlated with immunological status and sensitivity to anti-PD-1/PD-L1 therapy in gastric cancer

**Fig. 1:**
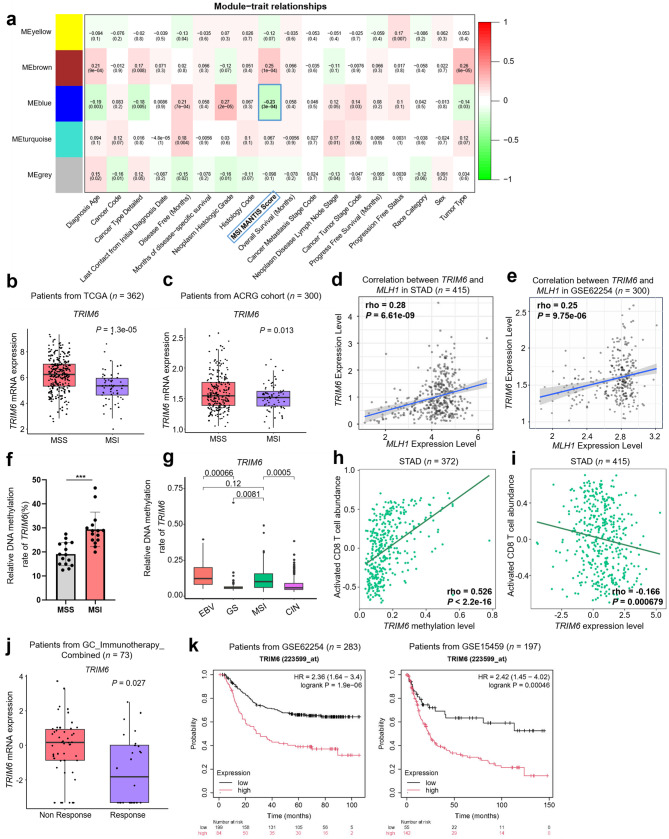
*TRIM6* expression correlates with immunological status and sensitivity to immunotherapy in GC. **a** Correlation between gene modules and clinical traits in the TCGA-STAD dataset was identified using WGCNA analysis. **b** and **c** Expression of *TRIM6* in MSI GC compared with MSS GC in TCGA-STAD (*n* = 362, *P* = 1.3e-05) and ACRG (*n* = 300, *P* = 0.013) cohorts. **d** and **e** Graph show the correlation of mRNA expression of *TRIM6* and *MLH1* in TCGA-STAD and GSE62254 cohorts. **f** CpG island methylation alterations in MSI and MSS GC from Zhongshan cohorts (*n* = 15 per group): bisulfite sequencing of 10 sites in *TRIM6* promoter (8 clones per cell line). The MSI status is determined by pathologists in Zhongshan Hospital through immunohistochemical evaluation of DNA mismatch repair proteins (MLH1, MSH2, MSH6, and PMS2) in tumor tissue sections. **g** DNA methylation status of *TRIM6* in different molecular subtypes of GC in TCGA-STAD cohort. **h** and **i** Correlation analysis between *TRIM6* methylation, and expression with activated CD8^+^ T cells infiltration in STAD by TISIB. **j** *TRIM6* expression in immunotherapy responders versus non-responders. Points represent log_2_(*TPM* + 0.1) values (*n* = 73). *P*-value was derived from a limma linear model (expression ~ Response_Group + Cohort), with cohort included as a covariate to adjust for batch effects. **k** Overall survival curves of *TRIM6* expression in patients with GC based on GSE62254 (*n* = 283) and GSE15459 (*n* = 197) databases. *p < 0.05, **p < 0.01, ***p < 0.001

On the basis of the TCGA-STAD dataset, which includes data from 374 gastric cancer patients, we performed weighted gene coexpression network analysis (WGCNA) [29] on more than 356 E3 ligases, including most members of the large E3 family, and analyzed their expression with the clinical features of gastric cancer patients (Fig. 1a, Supplementary Fig. 1a-c). Interestingly, coexpression analysis identified two modules (designated brown and blue) showing significant associations with the MSI MANTIS scores—a metric reflecting the MSI status of patient tumors [17] (Fig. 1a). Notably, the blue module demonstrated a strong negative correlation (*r* = -0.23, *P* = 3e-04) with MANTIS scores, suggesting its potential relevance as a therapeutic target. From the top 10 MANTIS-negatively-associated genes in this module, we identified *TRIM6* as the lead candidate due to its strongest negative correlation with overall survival (OS) in GC patients, implying its potential clinical relevance as a prognostic biomarker (Supplementary Fig. 1d). Therefore, we sought to investigate the role of TRIM6 in MSI and GC immunotherapy.

First, we validated the correlation of *TRIM6* expression with MSI-H or MSS in different gastric cancer datasets. Consistently, we found that *TRIM6* mRNA levels were significantly lower in MSI-H tumors from patients in the TCGA-STAD and ACRG cohorts (GSE62254) than in MSS tumors (Fig. 1b and c). Similarly, *TRIM6* expression was found to be positively associated with *MLH1* expression in gastric cancer (Fig. 1d and e). *MLH1* epigenetic silencing represents a typical molecular event and biomarker of MSI [30].

In addition, we found that compared with EBV-negative samples, EBV-positive gastric cancer samples presented lower *TRIM6* expression (Supplementary Fig. 1e). We next explored the mechanism underlying the relatively low level of TRIM6 in the immunological ‘hot’ subtypes of gastric cancer. EBV-CIMP (CpG island methylation phenotype) and MSI-associated gastric CIMP, which exhibit distinct methylation profiles, contribute to the epigenetic silencing of *CDKN2A* and *MLH1*, respectively [2]. Therefore, we sought to examine the DNA methylation levels of the *TRIM6* promoter in different GC subtypes. An analysis via Rajvir Dahiya’s algorithm revealed that the promoter region of *TRIM6* contains a CpG island from -851 to -702 of the transcription start site (TSS) [31] (Supplementary Fig. 1f). By using bisulfite sequencing, we found that, compared with that in MSS tumor tissues, *TRIM6* promoter methylation was significantly increased in MSI-H gastric tumor samples (Fig. 1f), which was verified with data from the TCGA-STAD methylation database (Fig. 1g).

Considering the immunological ‘hot’ features of MSI-H and EBV-positive GC and the downregulation of TRIM6 in these samples, we next analyzed whether TRIM6 expression is correlated with the immunological activity of GC. Interestingly, *TRIM6* methylation was positively correlated with the proportions of CTLs and activated CD8^+^ T-cell infiltration in GC (Supplementary Fig. 1g-i, Fig. 1h). Similarly, *TRIM6* expression was inversely correlated with the proportions of activated CD8^+^ T cells (Fig. 1i). Furthermore, gastric cancer patients with low *TRIM6* expression were more sensitive to anti-PD-1 therapy (Fig. 1j), with a better survival outcome (Fig. 1k). This inverse correlation between *TRIM6* and anti-PD-1 response was further confirmed in highly immunogenic melanoma [32] (Supplementary Fig. 1j), supporting TRIM6’s conserved role in immunotherapy regulation. Taken together, these analyses suggest that TRIM6 is correlated with the immunological status and sensitivity to anti-PD-1 treatment in GC patients.

### TRIM6 confers resistance to ICB therapy

Next, we investigated the immunogenic role of TRIM6 in two different mouse gastric cancer models: the MTC [33] and MFC [34] syngeneic subcutaneous tumor models. The MTC cell lines, harboring concurrent *Trp53* and *Cdh1* deletion, were generated from *Trp53*^fl/fl^; *Cdh1*^fl/fl^; *Mist1*-Cre mice that recapitulate the pathological features of diffuse gastric cancer (DGC). Importantly, clinical-pathological evaluation demonstrated that patients with genomically stable gastric cancer—characterized by *CDH1* loss and predominantly manifesting as DGC—showed significantly attenuated responses to immunotherapy [2, 35, 36].

**Fig. 2:**
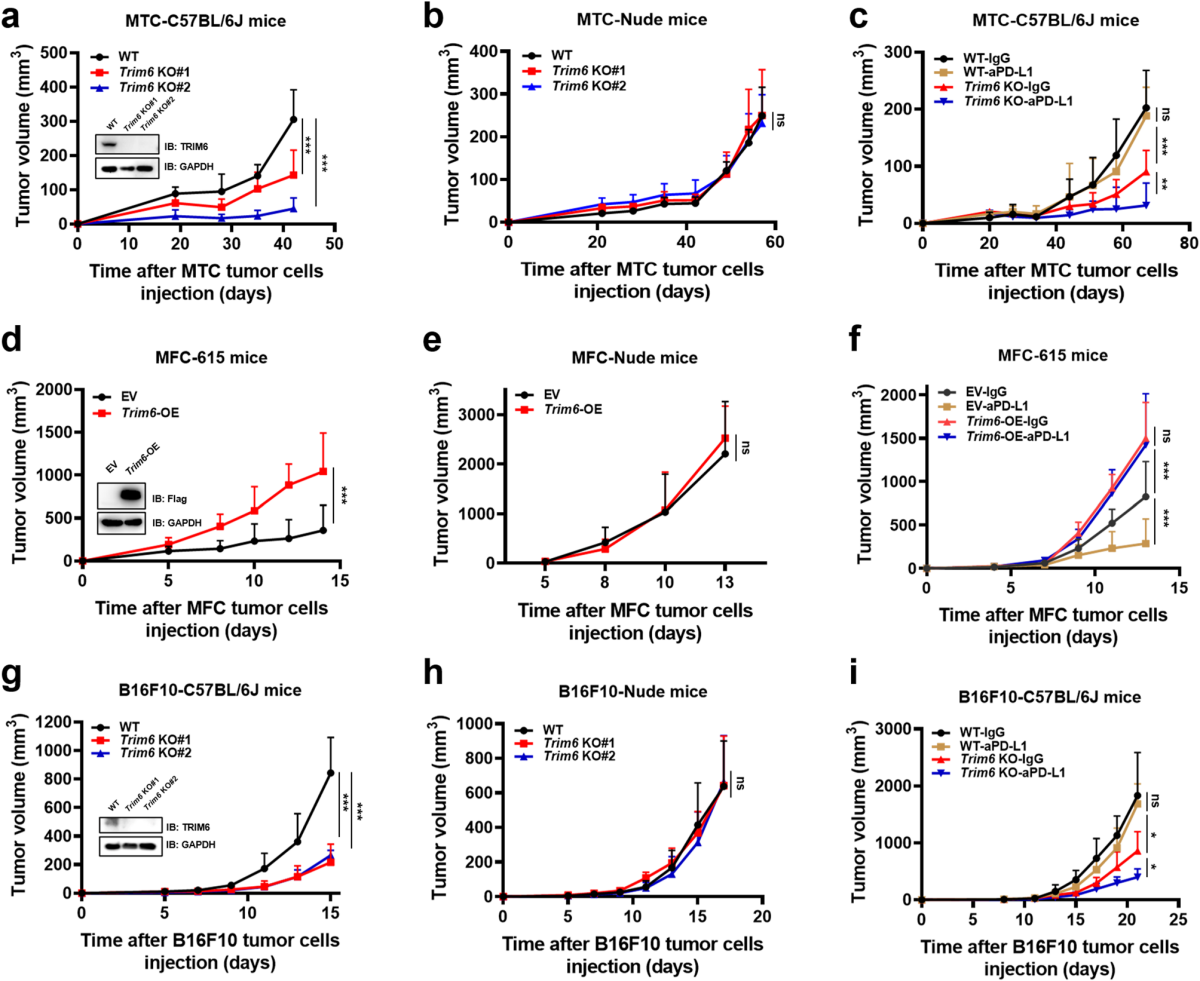
Knockout of *TRIM6* retards tumor growth and enhances tumor sensitivity to immunotherapy. **a-c** Tumor growth curves of C57BL/6J mice (*n* = 5) (**a**) or BALB/c nude mice (*n* = 5) (**b**) subcutaneously injected with 4 × 10^6^ WT or *Trim6* KO MTC cells and treated with 200 µg/mouse anti-PD-L1 or isotype control antibodies twice weekly, starting at 4 weeks post-inoculation (*n* = 5) (**c**). *Trim6* knockout efficiency in MTC cells was assessed by western blotting (anti-Trim6 antibody). **d-f** Tumor growth curves of 615 mice (*n* = 5) (**d**) or BALB/c nude mice (*n* = 6) (**e**) after subcutaneous injection of 2 × 10^6^ control or *Trim6* overexpression MFC cells and treatment with 200 µg/mouse anti-PD-L1 or isotype control antibodies on Days 6, 9 and 12 post-inoculation (*n* = 5) (**f**). *Trim6* overexpression efficiency in MFC cells was assessed by western blotting (anti-Flag antibody). **g-i** Tumor growth curves of C57BL/6J mice (*n* = 5) (**g**) and BALB/c nude mice (*n* = 5) (**h**) after subcutaneous injection of 1 × 10^5^ WT (*n* = 5 in IgG group; *n* = 4 in anti-PD-L1 group) or *Trim6* KO (*n* = 4) B16F10 cells and treatment with 200 µg/mouse anti-PD-L1 or isotype control antibodies twice weekly, starting at 7 Days post-inoculation (**i**). *Trim6* knockout efficiency in B16F10 cells was assessed by western blotting (anti-Trim6 antibody). ns, no significance; *p < 0.05, **p < 0.01, ***p < 0.001

We depleted *TRIM6* in MTC cells and observed the tumorigenesis of MTC cells in immune-competent and immune-deficient mice. As shown in Fig. 2a-b and Supplementary Fig. 2a-b, depletion of *TRIM6* retarded the tumor growth of MTC cells in immunocompetent C57BL/6J mice, whereas this suppression was compromised in nude mice.

Moreover, *TRIM6* depletion sensitized MTC tumors to anti-PD-L1 antibody treatment in C57BL/6J mice (Fig. 2c, Supplementary Fig. 2c). In contrast, the MFC tumor model, notable for its low degree of differentiation, poor intercellular adhesion, high invasiveness and strong metastatic potential [34], has been reported to be sensitive to anti-PD-L1 antibody treatment [37, 38]. The overexpression (OE) of *TRIM6* enhanced the tumorigenesis of MFC cells in immunocompetent 615 mice but not in nude mice (Fig. 2d-e, Supplementary Fig. 2d-e). *TRIM6*-OE also conferred MFC cell resistance to anti-PD-L1 treatment in 615 mice (Fig. 2f, Supplementary Fig. 2f). Moreover, we also found that *TRIM6* depletion inhibited the growth of B16F10 melanoma tumor in syngeneic mice and increased their response to anti-PD-L1 treatment (Fig. 2g-i, Supplementary Fig. 3a-c). Collectively, these data suggest that TRIM6 functions as a critical regulator of ICB resistance in gastric cancer, and genetic ablation of *TRIM6* substantially sensitizes gastric tumors to anti-PD-L1 therapy.

### Depletion of *TRIM6* increases the infiltration of CD8^+^ T lymphocytes through activating the innate immune response in gastric cancer

**Fig. 3:**
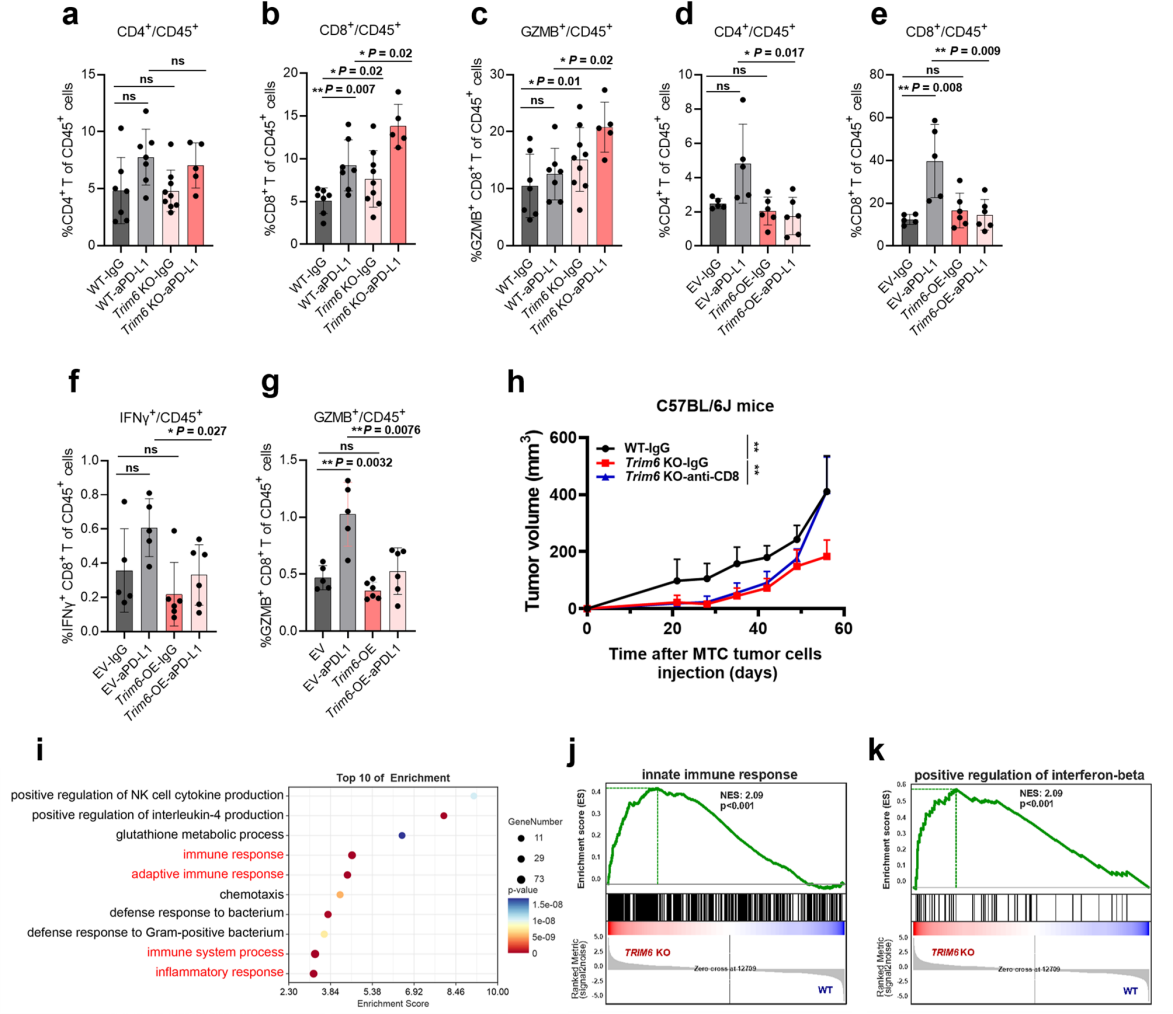
Deletion of *TRIM6* activates innate immune signaling and increases CD8^+^ T-cell infiltration. **a-c** Proportions of tumor-infiltrating CD4^+^ T cells (a), CD8^+^ T cells (b), and GZMB^+^CD8^+^ T cells (c) in tumor tissue from vector control (*n* = 7) or *Trim6* KO (*n* = 9 in IgG group; *n* = 5 in anti-PD-L1 group) MTC tumors derived from C57BL/6J mice. Mice received intraperitoneal injections of anti-PD-L1 (200 µg/mouse) or isotype control antibodies twice weekly from Week 4 to 6 post-inoculation. **d-g** Proportions of CD4^+^ T cells (d), CD8^+^ T cells (e), IFN-γ^+^ CD8^+^ T cells (f), and GZMB^+^ CD8^+^ T cells (g) in tumor tissue from vector control (*n* = 5) or *Trim6* OE (*n* = 6) MFC tumors derived from 615 mice. Mice received intraperitoneal injections of anti-PD-L1 (200 µg/mouse) or isotype control antibodies on Days 6, 9 and 12 post-inoculation. **h** Tumor growth curves of C57BL/6J mice subcutaneously injected with 2 × 10^6^ control (*n* = 6) or *Trim6* KO (*n* = 6) MTC cells, and treated with 200 µg/mouse anti-CD8 or isotype control antibodies on Days 26, 33, 40 and 47 after inoculation. **i** Top 10 enriched GO (biological process) terms of upregulated differentially expressed genes in *Trim6* KO MTC tumors (biological processes ranked by enrichment score; *n* = 4 biological replicates). **j** and **k** GSEA revealed an upregulated innate immune response (j) and positive regulation of the interferon-beta production pathway (k) in *Trim6* KO MTC tumors. ns, no significance; *p < 0.05, **p < 0.01, ***p < 0.001

To investigate the mechanism by which TRIM6 regulates sensitivity to ICB therapy in gastric cancer, we analyzed tumor-infiltrating lymphocytes via flow cytometry using two mouse tumor models under anti-PD-L1 treatment: MTC (with *Trim6* depletion), and MFC (with *Trim6* overexpression). As shown in Fig. 3a, b, and c, deletion of *Trim6* in combination with anti-PD-L1 treatment increased the proportions of CD8^+^ and CD8^+^ GZMB^+^ T cells but not those of CD4^+^ T cells in MTC tumors. In contrast, *Trim6* overexpression decreased effective CD8^+^ T-cell infiltration in MFC tumors under anti-PD-L1 treatment (Fig. 3d-g). These results suggested that TRIM6 regulation of gastric tumor immunity might be mediated by CD8^+^ T cells. Consistent with this speculation, blocking CD8^+^ T cells with an anti-CD8 antibody significantly reversed the inhibition of tumor growth in the *Trim6* KO MTC model (Fig. 3h, Supplementary Fig. 4a).

Next, we performed transcriptomic profiling of WT and *Trim6* KO MTC tumors using RNA-sequencing. GO enrichment analysis revealed enrichment of multiple pathways related to the immune response and inflammatory response pathways when *Trim6* was depleted (Fig. 3i). GSEA revealed that the innate immune response and positive regulation of the interferon-beta production pathway were enriched in *Trim6* KO MTC tumors (Fig. 3j-k, Supplementary Fig. 4b-c). These data suggest that TRIM6 may regulate antitumor immunity and immunotherapy sensitivity by affecting the innate immune response.

### TRIM6 triggers the proteasomal degradation of cGAS via K27-linked polyubiquitination

**Fig. 4:**
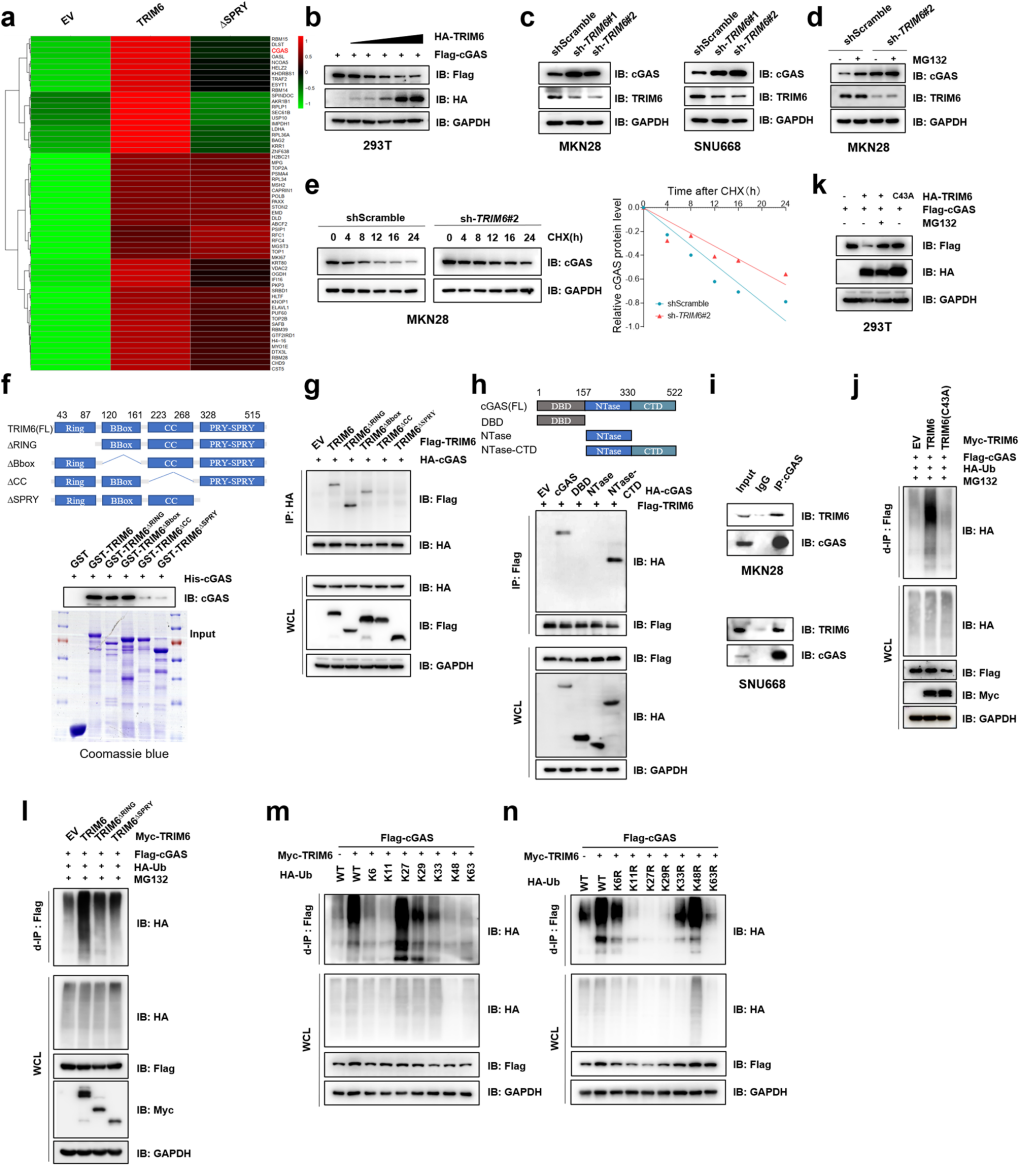
TRIM6 mediates proteasomal degradation of cGAS through K27-linked ubiquitination. **a** Heatmap depicting protein enrichment profiles from Flag-tagged TRIM6 full length and ΔSPRY mutant co-immunoprecipitation in MKN28 gastric cancer cells. **b** IB analysis of 293T cells co-transfected with constant Flag-cGAS (1 µg) and gradient HA-TRIM6 plasmids. **c** IB analysis of cGAS, TRIM6 proteins in MKN28 and SNU668 gastric cancer cells with shScramble control or sh-*TRIM6* knockdown. **d** cGAS protein accumulation upon MG132 treatment in MKN28-shScramble cells. **e** cGAS protein was measured by IB analysis in MKN28-shScramble and MKN28-sh-*TRIM6* cells treated with 100 µg/mL CHX at the indicated time points. **f** GST pull-down analysis of purified His-cGAS incubated with GST (control) or GST-TRIM6 and its truncated mutants. **g** Immunoprecipitation (with anti-HA) and IB analysis of HEK293T cells transfected with HA-cGAS and Flag-TRIM6 or its truncated mutants for 48 h. **h** Immunoprecipitation (with anti-Flag) and IB analysis of HEK293T cells transfected with Flag-TRIM6 and HA-cGAS or its truncated mutants for 48 h. **i** Endogenous co-IP analysis in MKN28 and SNU668 cells were performed using an anti-cGAS antibody. Rabbit IgG was used as a control. **j** Denature-IP (with anti-Flag) and IB analysis of HEK293T cells transfected with Flag-cGAS, HA-Ub, Myc-TRIM6 (WT/C43A), and treated with MG132 for 12 h. **k** IB analysis of HEK293T cells transfected with Flag-cGAS, HA-TRIM6 (WT/C43A), and treated with MG132 for 12 h. **l** Denature-IP (with anti-Flag) and IB analysis of HEK293T cells transfected with Flag-cGAS, HA-Ub, Myc-TRIM6 and its truncated mutants, and treated with MG132 for 12 h. **m** and **n** Denature-IP (with anti-Flag) and immunoblot analysis of HEK293T cells transfected with Flag-cGAS, Myc-TRIM6 and HA-Ub with K-only (m) or K-to-R (n) mutates, and treated with MG132 for 12 h

To clarify the underlying mechanism by which TRIM6 regulates gastric tumor immunotherapy, we conducted immunoprecipitation using anti-Flag beads in MKN28 gastric cancer cell line transfected with Flag-tagged TRIM6 or a truncation mutant with deletion in the PRY-SPRY domain, which are responsible for TRIM6 substrate binding [39] (Supplementary Fig. 5a). The IP-MS results revealed that cGAS, OASL, USP10, DLST, KHDRBS1 and ZNF638 were immunoprecipitated by wild-type TRIM6 but not its PRY-SPRY domain-truncated mutants (ΔSPRY), among which the pivotal role of cGAS in antiviral defense and antitumor immunity attracted our attention (Fig. 4a). We therefore investigated the biochemical regulatory effects of TRIM6 on cGAS and cGAS signaling.

As shown in Fig. 4b, TRIM6 attenuated cGAS protein abundance in a dose-dependent manner in 293T cells. In the gastric cancer cell lines MKN28 and SNU668, both of which are MSS or MSI-low [40], we knocked down *TRIM6* and found that the cGAS protein level was elevated in sh*TRIM6* cells, whereas their mRNA expression remained unaffected (Fig. 4c, Supplementary Fig. 5b). The protein abundance of cGAS increased after MG132 treatment, and the half-life of cGAS was prolonged in sh*TRIM6* cells (Fig. 4d and e).

TRIM6 contains an N-terminal tripartite RBCC motif (RING, B-box, coiled-coil) and a C-terminal PRY-SPRY domain [39]. The RING domain is recognized for its E3 ubiquitin ligase activity; the CC domain is necessary for the ability of TRIM family proteins to form homo or hetero-oligomers; and the PRY-SPRY domain mediates substrate interactions [41, 42]. GST pull-down and co-IP analyses revealed that cGAS could interact with full-length or ΔRING/ΔBOX TRIM6, whereas their interaction was abolished by deletion of the CC domain or PRY-SPRY domain in TRIM6 (Fig. 4f and g). The structure of cGAS includes an N-terminal DNA-binding domain (DBD), a central nucleotidyltransferase (NTase) domain, and a C-terminal domain (CTD) [43]. Our domain mapping analysis suggested that the C-terminal region of cGAS but not its N-terminal region (DBD) was responsible for the TRIM6-cGAS association (Fig. 4h). Moreover, endogenous co-IP confirmed the interaction between TRIM6 and cGAS in gastric cancer cell lines (Fig. 4i). Immunofluorescence staining revealed that TRIM6 colocalized with cGAS in the cytostome (Supplementary Fig. 5c).

We subsequently investigated the role of TRIM6 in the ubiquitination status of cGAS. The TRIM6 C43A (TRIM6 C15A in the short form) catalytically inactivated mutant lost the ability to synthesize poly-Ub chains within substrates [44]. We found that TRIM6, but not its C43A catalytically inactive mutant, remarkably promoted the polyubiquitination of cGAS (Fig. 4j). As a result, the catalytically inactive TRIM6 mutant failed to degrade cGAS in 293T cells (Fig. 4k). Additionally, the two TRIM6 truncation mutants with deletions in the RING or SPRY domains did not ubiquitinate cGAS (Fig. 4l). We next performed ubiquitination analysis by employing different ubiquitin proteins with K-to-R or K-only mutations. The results showed that TRIM6 robustly activated the K27-linked ubiquitination of cGAS, which was completely abrogated by the linkage of K27R ubiquitin (Fig. 4m and n). Therefore, our data suggested that TRIM6 triggers the proteasomal degradation of cGAS via K27-linked polyubiquitination and negatively regulates cGAS abundance in gastric cancer cells.

### TRIM6 negatively regulates cGAS-STING-stimulated type I IFN signaling

**Fig. 5:**
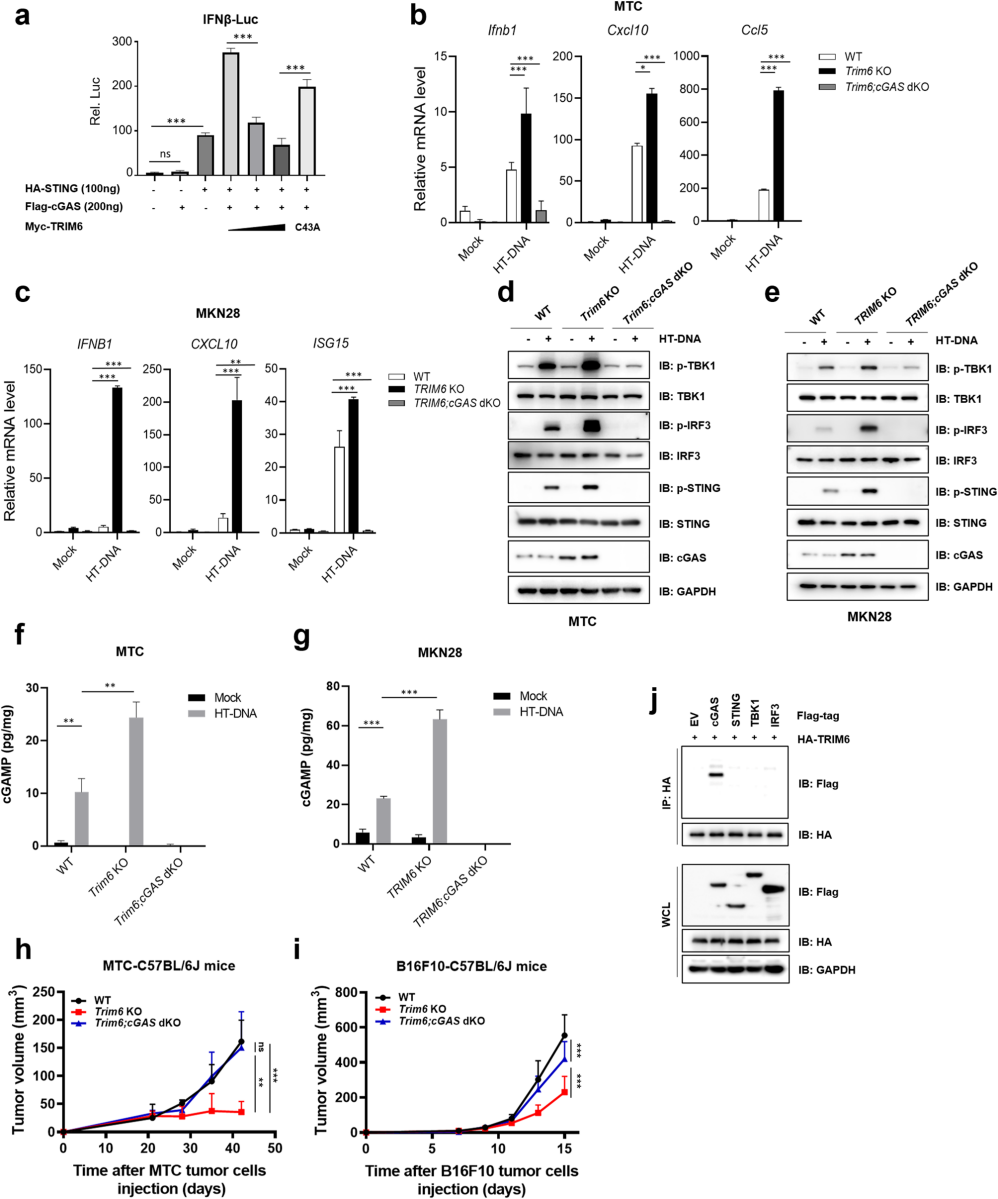
TRIM6 suppresses cGAS-STING-driven type I interferon signaling. **a** Luciferase reporter assays in HEK293T cells co-expressing Flag-cGAS, HA-STING, and Myc-TRIM6 (WT/C43A). **b** and **c** RT-qPCR analysis of *Ifnb1*, *Cxcl10* and *Ccl5* or *ISG15* mRNA in WT, *TRIM6* KO and *TRIM6; cGAS* dKO MTC (**b**) and MKN28 cells (**c**) transfected with HT-DNA (2 µg/mL) for 6 h. **d** and **e** IB analysis of WT, *TRIM6* KO and *TRIM6; cGAS* dKO MTC (**d**) and MKN28 cells (**e**) transfected with HT-DNA (2 µg/mL) for 6 h. **f** and **g** cGAMP levels measured by ELISA in WT, *TRIM6* KO, and *TRIM6; cGAS* dKO MTC (**f**) and MKN28 (**g**) cells transfected with HT-DNA (2 µg/mL) for 12 h. **h** and **i** Tumor growth curves of C57BL/6J mice subcutaneously injected with WT, *Trim6* KO and *Trim6; cGAS* dKO MTC (**h**) and B16F10 cells (**i**). **j** Immunoprecipitation (with anti-HA) and IB analysis of HEK293T cells transfected with HA-TRIM6 and Flag-cGAS, Flag-STING, Flag-TBK1, and Flag-IRF3 for 48 h. ns, no significance; *p < 0.05, **p < 0.01, ***p < 0.001

Next, we investigated whether TRIM6 is involved in the regulation of cGAS-mediated immune signaling. We transfected Flag-cGAS, HA-STING, and Myc-TRIM6 or its catalytically inactivated mutant TRIM6 (C43A) into the IFNβ luciferase reporter system. The luciferase assay results revealed that TRIM6 significantly inhibited the activation of the cGAS-STING-activated IFNβ signal in a dose-dependent manner, whereas TRIM6 (C43A) failed to do so (Fig. 5a). Conversely, *TRIM6* depletion in mouse (MTC) and human (MKN28) gastric cancer cells resulted in a significant increase in the mRNA levels of *IFNB1* and its downstream genes *CXCL10* and *ISG15* (or *Ccl5*) under HT-DNA treatment, and these effects were abolished by simultaneous *cGAS* knockout (Fig. 5b and c). In addition, the levels of phosphorylated STING, TBK1 and IRF3 (three crucial mediators downstream of the cGAS-STING signaling axis) proteins were markedly increased in three distinct *TRIM6* KO cell lines (MTC, MKN28, and SNU668) but were abrogated upon *cGAS* depletion (Fig. 5d and e, Supplementary Fig. 6a).

To further support the activated cGAS signal in *TRIM6* KO cells, we examined the synthesis of cGAMP in MTC and MKN28 cells and found that cGAMP was robustly elevated when *TRIM6* was depleted, which was completely abrogated in *TRIM6; cGAS* double knockout (dKO) cells (Fig. 5f and g). These results suggest that TRIM6 regulates cGAS-STING-IFNβ signaling in a cGAS-dependent manner. Meanwhile, *Trim6; cGAS* dKO cells substantially abrogated the tumor growth suppressive effect observed in *Trim6* KO cells when MTC and B16F10 cells were transplanted into C57BL/6J mice (Fig. 5h-i and Supplementary Fig. 6b-c). Furthermore, co-IP analysis revealed that TRIM6 does not physically interact with STING, TBK1, or IRF3 (Fig. 5j). These data suggest that TRIM6 attenuates cGAS-STING-dependent type I interferon responses through the degradation of cGAS, which facilitates tumor immune evasion and accelerates tumor progression.

### Conditional knockout of *Trim6* suppresses gastric tumorigenesis in mouse models with *Trp53* knockout and *Myc* overexpression

**Fig. 6:**
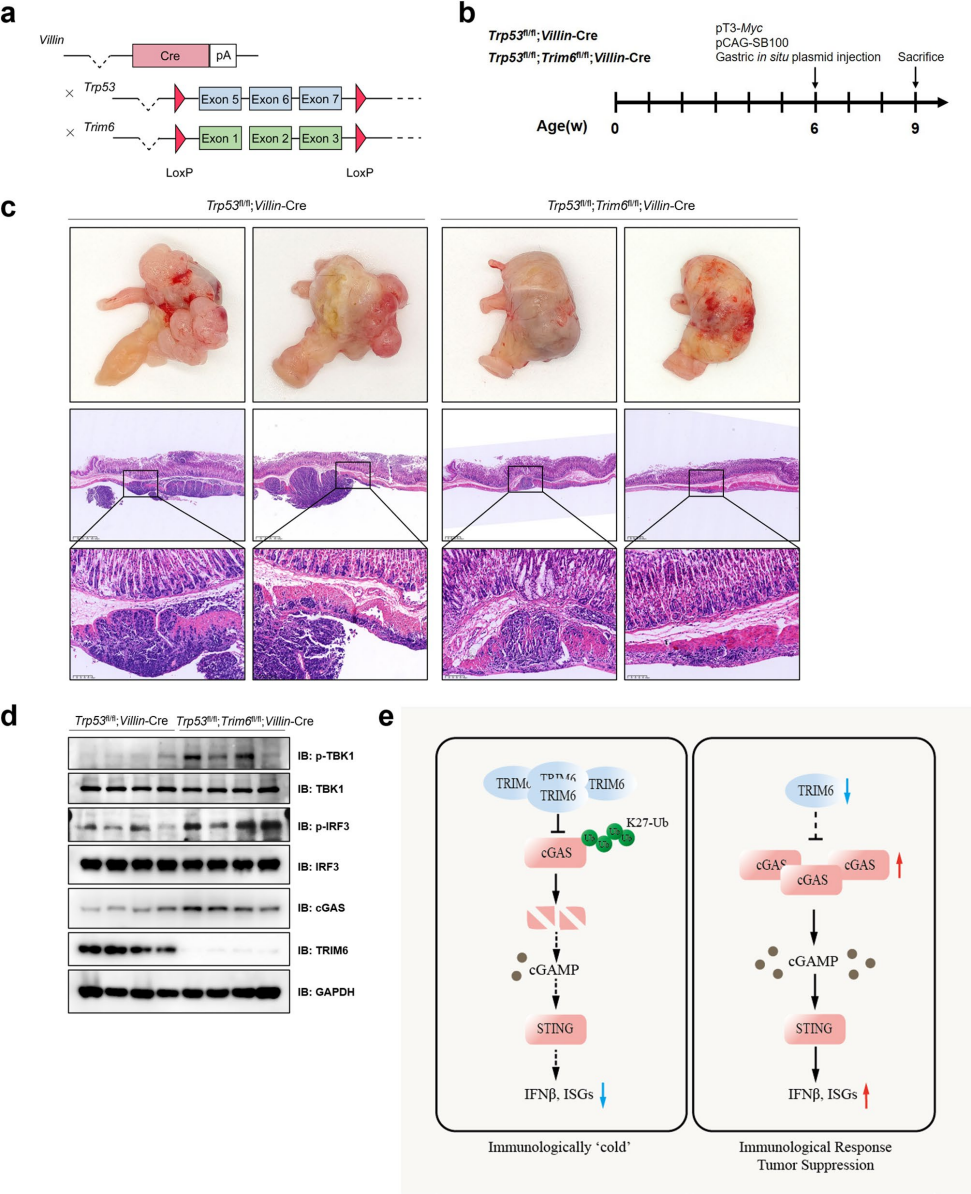
TRIM6 suppresses cGAS-STING signaling in a mouse transgenic gastric cancer model. **a** Schematic illustration of the generation of mice with distinct genotypes. **b** Scheme for the generation of transgenic gastric cancer mouse model. **c** Gross morphology and H&E staining of gastric tumors in *Trp53*^fl/fl^; *Villin*-Cre mice versus *Trp53*^fl/fl^; *Trim6*^fl/fl^; *Villin*-Cre mice (*n* = 5 per group). Scale bars: 400 μm and 100 μm. **d** IB analysis of TRIM6, cGAS, p-TBK1, and p-IRF3 proteins in mouse gastric cancer tissues. **e** Schematic representation of targeting TRIM6 for activating cGAS-STING-IFNβ signaling and potentiating antitumor immune responses in GC

To investigate whether TRIM6 regulates cGAS-STING signaling and gastric tumorigenesis in vivo, we generated *Trp53*^fl/fl^; *Villin-*Cre *and Trp53*^fl/fl^; *Trim6*^fl/fl^; *Villin-*Cre conditional knockout mice (Fig. 6a). Mouse genotypes were validated by polymerase chain reaction (PCR) analysis (Supplementary Fig. 7a). *Villin-*Cre*-*mediated recombination enables gene ablation in the gastric and intestinal epithelia [45]. Previous evidence indicates that *Myc* amplification and *Trp53* knockout synergistically induced the formation of MSS-type gastric tumors [46]. Therefore, we delivered the plasmids pT3-*Myc* and pCAG-SB100 into the gastric epithelium of *Trp53*^fl/fl^; *Villin-*Cre or *Trp53*^fl/fl^; *Trim6*^fl/fl^; *Villin-*Cre mice via in situ injection into stomach (Fig. 6b). Three weeks later, we collected gastric tissues and observed in situ gastric carcinogenesis in these mouse cohorts. Interestingly, *Trim6* depletion significantly attenuated the gastric tumorigenesis of mouse models (*Myc*^OE^; *Trp53*^−/−^) induced by *Trp53* deficiency coupled with *Myc* overexpression (Fig. 6c). This phenomenon aligns with our previous findings in the MTC (*Trp53*^*−/−*^; *Cdh1*^*−/−*^) gastric cancer model, where *Trim6* knockout similarly suppressed the tumor growth of MTC cells in C57BL/6J mice (Fig. 2a), reinforcing the role of TRIM6 across different MSS-type gastric tumor models. Furthermore, immunoblotting revealed increased activation of the cGAS-STING pathway in *Trim6*-depleted tissues, as evidenced by elevated protein levels of cGAS, phosphorylated TBK1 (p-TBK1), and phosphorylated IRF3 (p-IRF3) (Fig. 6d). These data indicate that *Trim6* loss triggers innate immune signaling via cGAS-STING in vivo, suggesting a potential mechanism by which *Trim6* deficiency enhances immune-mediated suppression of gastric tumorigenesis.

In summary, our study demonstrates that elevated TRIM6 characterizes MSS gastric cancers and inversely correlates with MSI status. Clinically, high TRIM6 levels associate with reduced CTLs infiltration and poorer response to pembrolizumab therapy. Mechanistically, we establish that TRIM6 mediates K27-linked polyubiquitination and subsequent proteasomal degradation of cGAS, effectively suppressing innate immune signaling. Through genetic ablation studies in both MSS/MSI-low gastric cancer cell lines and murine MSS models, we show that TRIM6 deletion restores cGAS protein stability, reactivates the cGAS-STING-IFNβ signaling axis (Fig. 6e), and enhances antitumor immunity. These findings suggest that TRIM6 targeting could potentially convert immunologically cold gastric tumors into immunotherapy-responsive hot phenotypes.

## Discussion

The sensitivity of MSI-H tumors to ICB has long been attributed to their hypermutated phenotype, which leads to the generation of abundant neoantigens [47, 48]. However, a recent study demonstrated that activation of the cGAS-STING pathway, not the tumor mutational burden (TMB), is the critical determinant of T-cell priming and ICB efficacy in MSI-H tumors. *cGAS* deficiency abolishes ICB responsiveness even in MSI models, revealing a TMB-independent mechanism underlying treatment resistance [49]. The cGAS-STING pathway has garnered significant attention for its role as a cytosolic DNA sensor within the innate immune response and therapeutic effect in enhancing antiviral defense and antitumor immunity [50–55]. Notably, the therapeutic efficacy of anti-PD-L1 treatment was found to be contingent upon cGAS expression, and no significant response was observed in tumors implanted in *cGAS*-deficient mice following PD-L1 blockade [56].

Here, we demonstrated that the RING E3 ligase TRIM6 can degrade cGAS via K27-linked polyubiquitination and impair cGAS-STING signaling in gastric cancer. Notably, *TRIM6* expression was originally found to be significantly associated with MSI status via WGCNA. *TRIM6* expression was significantly lower in immunologically ‘hot’ gastric cancers (MSI-H and EBV-positive groups) than in the other two immunologically ‘cold’ subtypes (GS and CIN groups). Conversely, elevated cGAS-STING signaling was identified in MSI-H and EBV-positive gastric tumors [57, 58]. In this study, we utilized MSS mouse gastric cancer MTC cells (*Trp53*^−/−^; *Cdh1*^−/−^), MSS gastric cancer in vivo models (*Myc*^OE^; *Trp53*^−/−^), and human MSS/MSI-low cell lines (SNU668, MKN28) for immunological and mechanistic investigations. Strikingly, *Trim6* deletion activated the cGAS-STING pathway and simultaneously inhibited tumor progression in MSS malignancies. *Trim6* depletion in immunologically ‘cold’ MSS gastric cancer cells promotes CD8^+^ T-cell infiltration by activating the cGAS-mediated innate immune response and sensitizes these tumors to anti-PD-1/PD-L1 treatment. Collectively, these findings suggest that TRIM6 might contribute to the immunological regulation of gastric cancer by degrading the cGAS protein.

Recent efforts to enhance immunotherapy efficacy in MSS tumors have primarily focused on colorectal cancer [59–61], with limited exploration of MSS gastric cancer. Our study identifies TRIM6 as a critical negative regulator of the cGAS-STING innate immune pathway in MSS gastric tumors, revealing a potential therapeutic vulnerability. Future research should prioritize the development of TRIM6-targeted agents and evaluate their safety and combinatorial efficacy with immune checkpoint blockade in preclinical MSS models.

## Conclusions

Our study reveals that the RING-type E3 ubiquitin ligase TRIM6 correlates with MSI and the immunological status of gastric cancer. *TRIM6* depletion significantly enhances CD8^+^ T-cell infiltration and sensitizes gastric tumors to anti-PD-1/PD-L1 treatment. Mechanistically, TRIM6 catalyzes the K27-linked polyubiquitination of cGAS, promoting its proteasomal degradation and subsequent inactivation of the cGAS-STING-IFNβ innate immune pathway. These findings position TRIM6 as both a novel predictive biomarker for immunotherapy response and a promising therapeutic target for immunologically cold gastric cancers.

## Supplementary Information

Below is the link to the electronic supplementary material.

Supplementary Material 1

## Data Availability

The raw RNA-seq data generated in this study have been deposited in the Genome Sequence Archive (GSA) at the National Genomics Data Center (NGDC), China National Center for Bioinformation / Beijing Institute of Genomics, Chinese Academy of Sciences, under accession number CRA025505. These data are publicly available at https://ngdc.cncb.ac.cn/gsa.

## Abbreviations

cGAS: Cyclic GMP-AMP synthase
CIMP: CpG island methylation phenotype
CTLs: Cytotoxic T lymphocytes
EBV: Epstein-Barr virus
GEO: Gene Expression Omnibus database
GO: Gene Ontology
ICB: Immune checkpoint blockade
IFN: Interferon
IP: Immunoprecipitation
MSI-H: Microsatellite instability-high
MSS: Microsatellite stable
OS: Overall survival
RNA-seq: RNA sequencing
STING: Stimulator of interferon genes
TCGA: The Cancer Genome Atlas
TRIM6: Tripartite motif containing 6
WGCNA: Weighted gene coexpression network analysis
