# Supplementary material for: TRIM6 ablation reverses ICB resistance in MSS gastric cancer by unleashing cGAS-STING-dependent antitumor immunity
Source: J Exp Clin Cancer Res. 2025 Aug 15;44:242. doi: 10.1186/s13046-025-03513-5 (PMC12355757; doi:10.1186/s13046-025-03513-5)
Supplement: Supplementary file 1 — Supplementary Material 1 [file 13046_2025_3513_MOESM1_ESM.docx]

**Supplementary Material**

**TRIM6 ablation reverses ICB resistance in MSS gastric cancer by unleashing cGAS-STING-dependent antitumor immunity**

**Niu *et al.***

**Supplementary Figures**

**
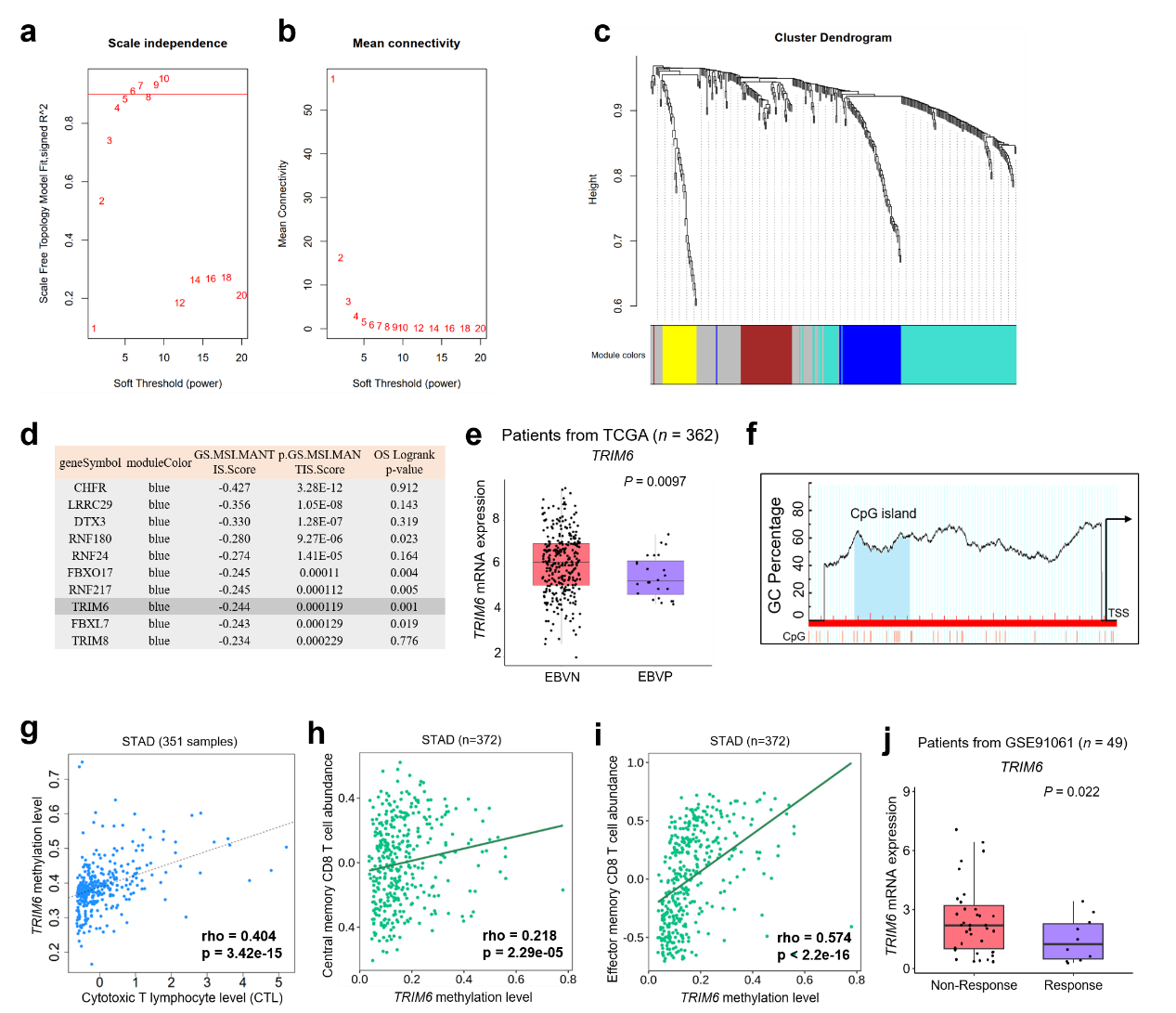
**

**Supplementary Figure S1. WGCNA analysis of the multiple E3 ligase genes in gastric cancer.**

**a** Screening of soft-thresholding powers. **b** Mean connectivity attenuation with increasing soft-threshold powers. **c** TOM-based hierarchical clustering reveals co-expression module dynamics before and after branch merging. **d** Ranking of genes in the blue co-expression module based on MSI scores, with integration of survival-associated signatures (Overall Survival [OS] Log-rank test *P*-values). **e** Expression of *TRIM6* in EBV^+^ GC compared to EBV^-^ GC from TCGA-STAD cohorts. **f** CpG island prediction in *TRIM6* promoter region (-851 to -702 from TSS). **g-i** Correlation analysis between *TRIM6* methylation and cytotoxic T lymphocytes (CTLs) (**g**), central memory CD8^+^ T cells (**h**), and effector memory CD8^+^ T cells (**i**) infiltration in STAD by TIDE and TISIB. **j** Expression of *TRIM6* in immunotherapy response and non-response groups in melanoma (GSE91061, *n* = 49, *P* = 0.022).

**
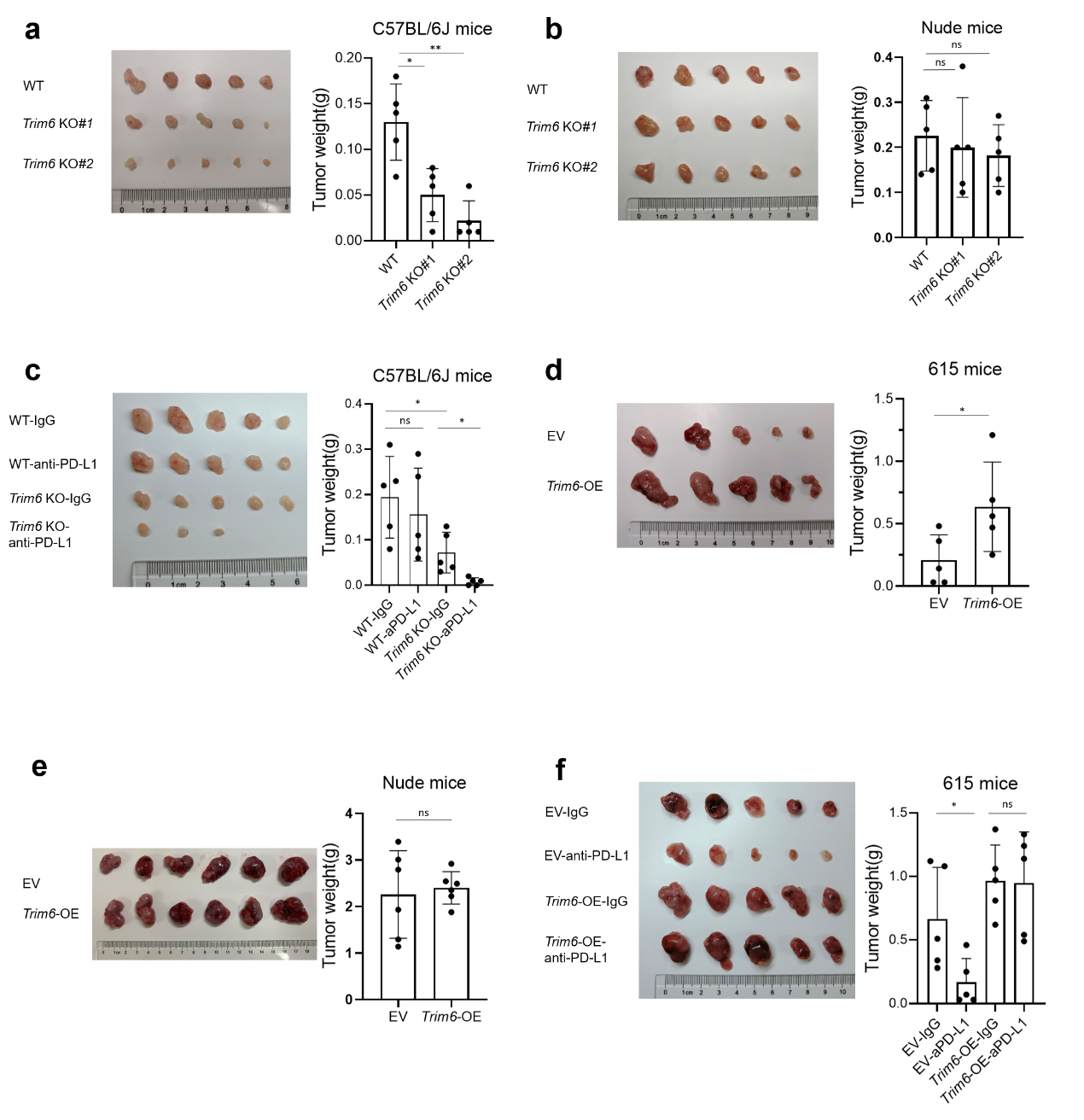
**

**Supplementary Figure S2. Tumorigenesis of mouse gastric cancer models with *Trim6* depletion or overexpression.**

**a-****c** Tumor growth and mass of WT and *Trim6* KO MTC cells in C57BL/6J (**a**) and nude (BALB/c) mice (**b**) following inoculation and treatment with anti-PD-L1 or isotype control antibodies (**c**). **d-f** Tumor growth and mass of WT and *Trim6* OE MFC cells in 615 (**d**) and nude (BALB/c) mice (**e**), following inoculation and treatment with anti-PD-L1 or isotype control antibodies (**f**). ns, no significance; **p* < 0.05, ***p* < 0.01, ****p* < 0.001

**
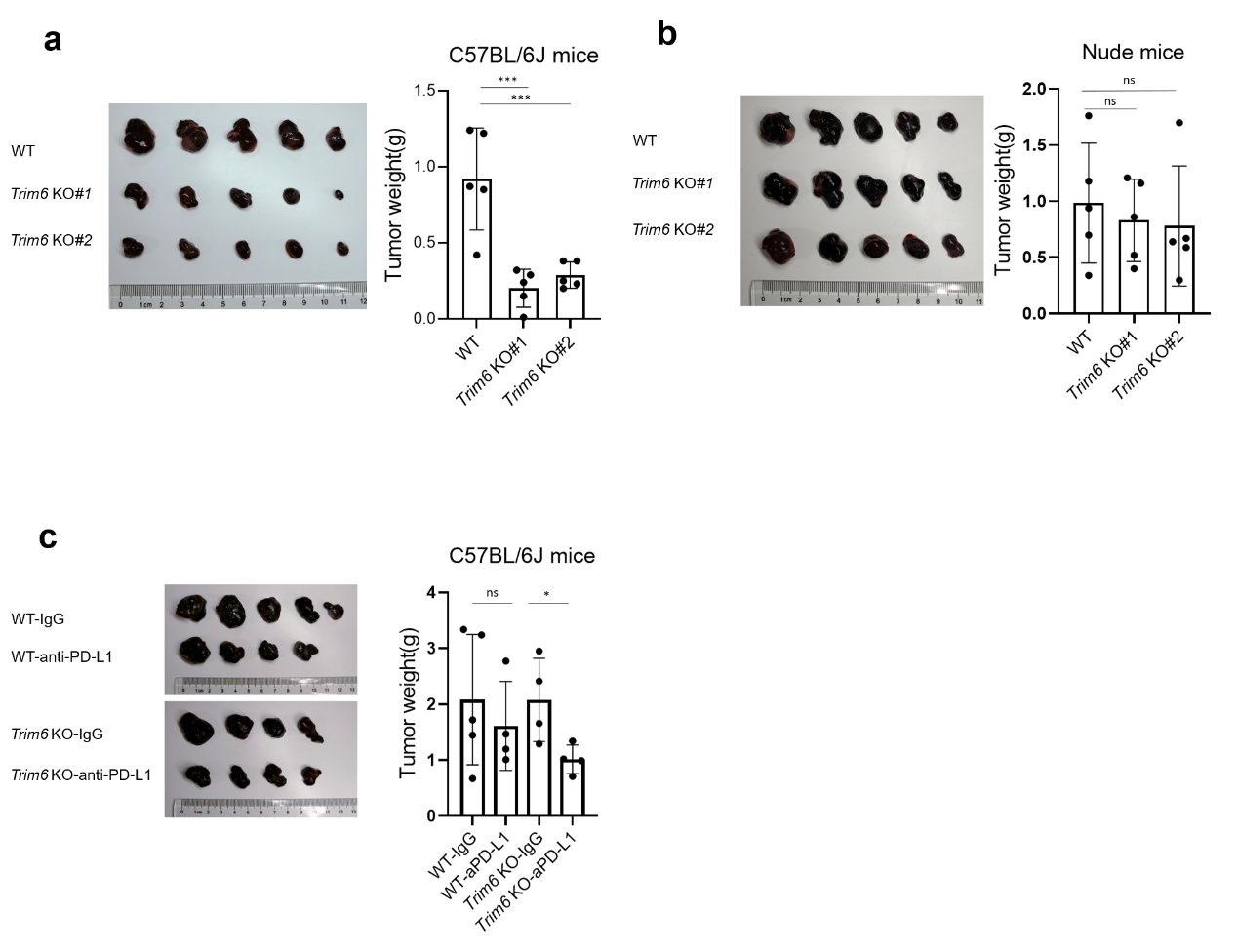
**

**Supplementary Figure S3. Tumorigenesis of mouse melanoma cancer models with *Trim6* depletion.**

**a-c** Tumor growth and mass of WT and *Trim6* KO B16F10 cells in C57BL/6J (**a**) and nude (BALB/c) mice (**b**) after inoculation and treatment with anti-PD-L1 or isotype control antibodies (**c**). WT tumors were collected on day 21 post-implantation, while *Trim6* KO tumors required extended monitoring and were harvested at day 24. ns, no significance; **p* < 0.05, ***p* < 0.01, ****p* < 0.001

**
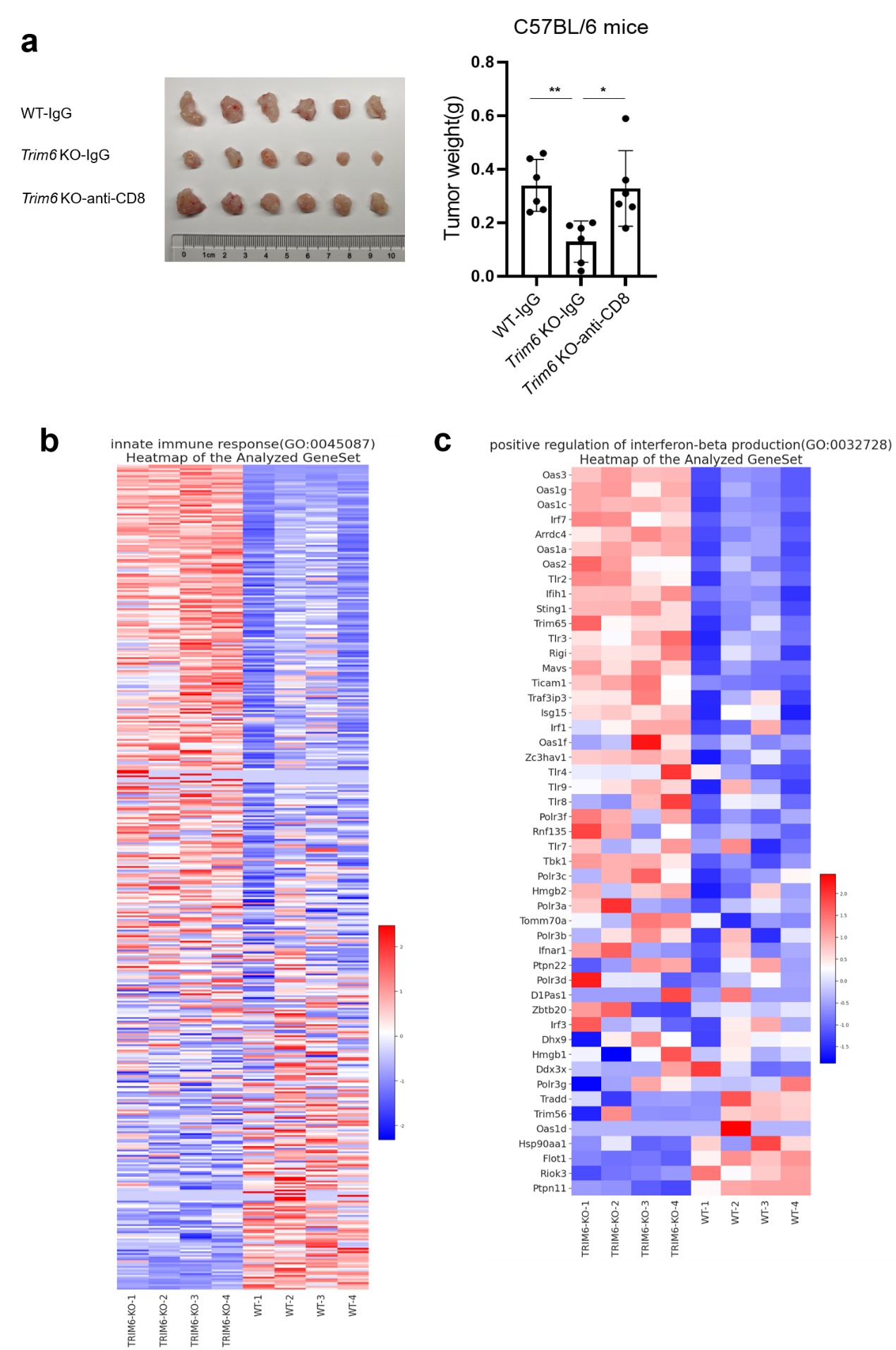
**

**Supplementary Figure S4. *Trim6* ablation activates innate immune responses.**

**a** Tumor growth and mass of WT and *Trim6* KO MTC tumors treated with anti-CD8 or isotype control antibodies. **b** and **c** Heatmap of genes enriched in the innate immune response (**b**) and the positive regulation of interferon-beta production pathway (**c**). **p* < 0.05, ***p* < 0.01, ****p* < 0.001


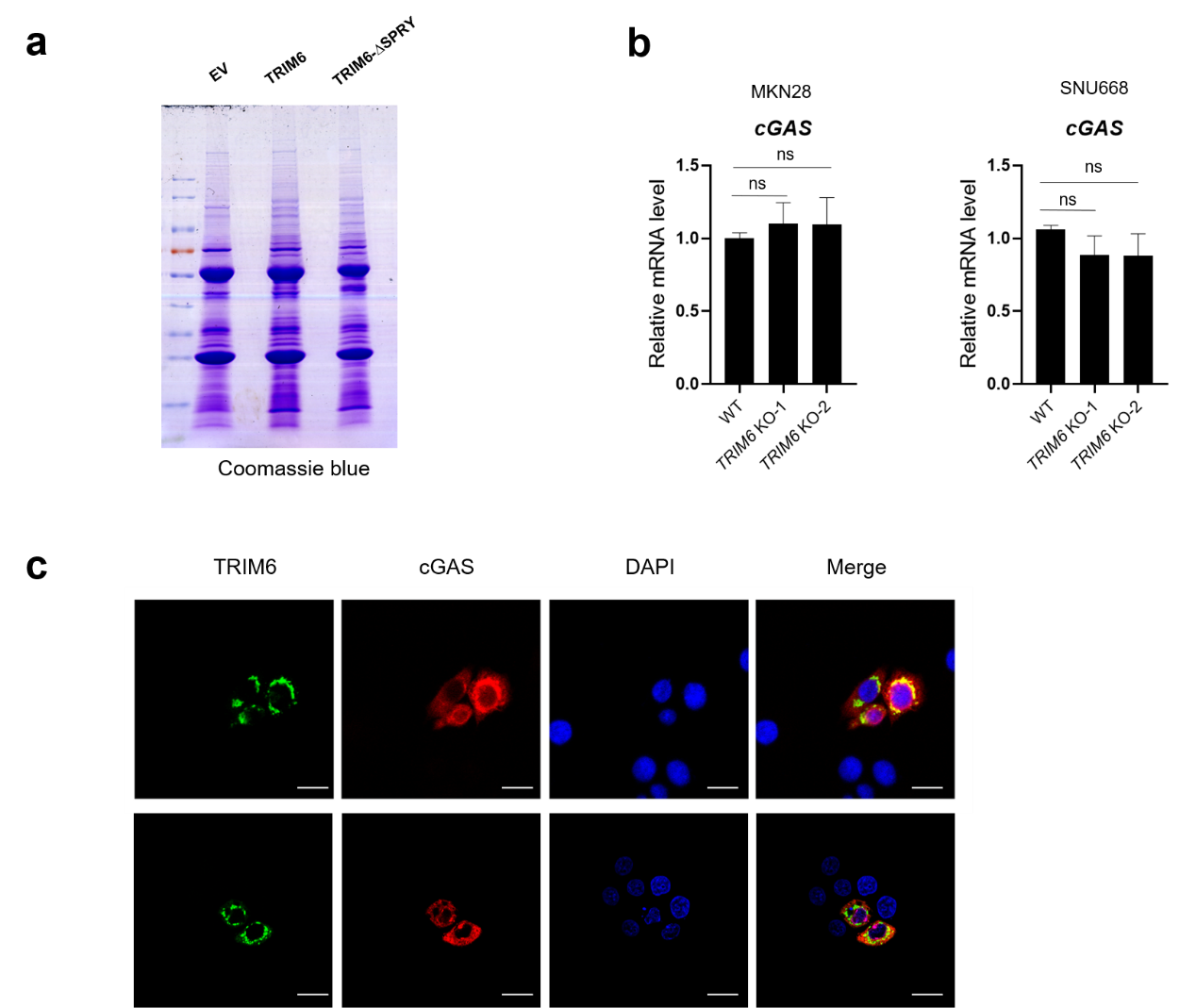


**Supplementary Figure S5. TRIM6 colocalizes with cGAS and does not affect *cGAS* mRNA expression.**

**a** Coomassie blue staining analysis of proteins immunoprecipitated with anti-Flag M2 beads. **b** RT-qPCR analysis of *cGAS* mRNA in WT and *TRIM6* KO MKN28 and SNU668 cells. **c** Cytoplasmic co-localization of TRIM6 and cGAS in MKN28 cells. Scale bar, 20 μm. ns, no significance; **p* < 0.05, ***p* < 0.01, ****p* < 0.001


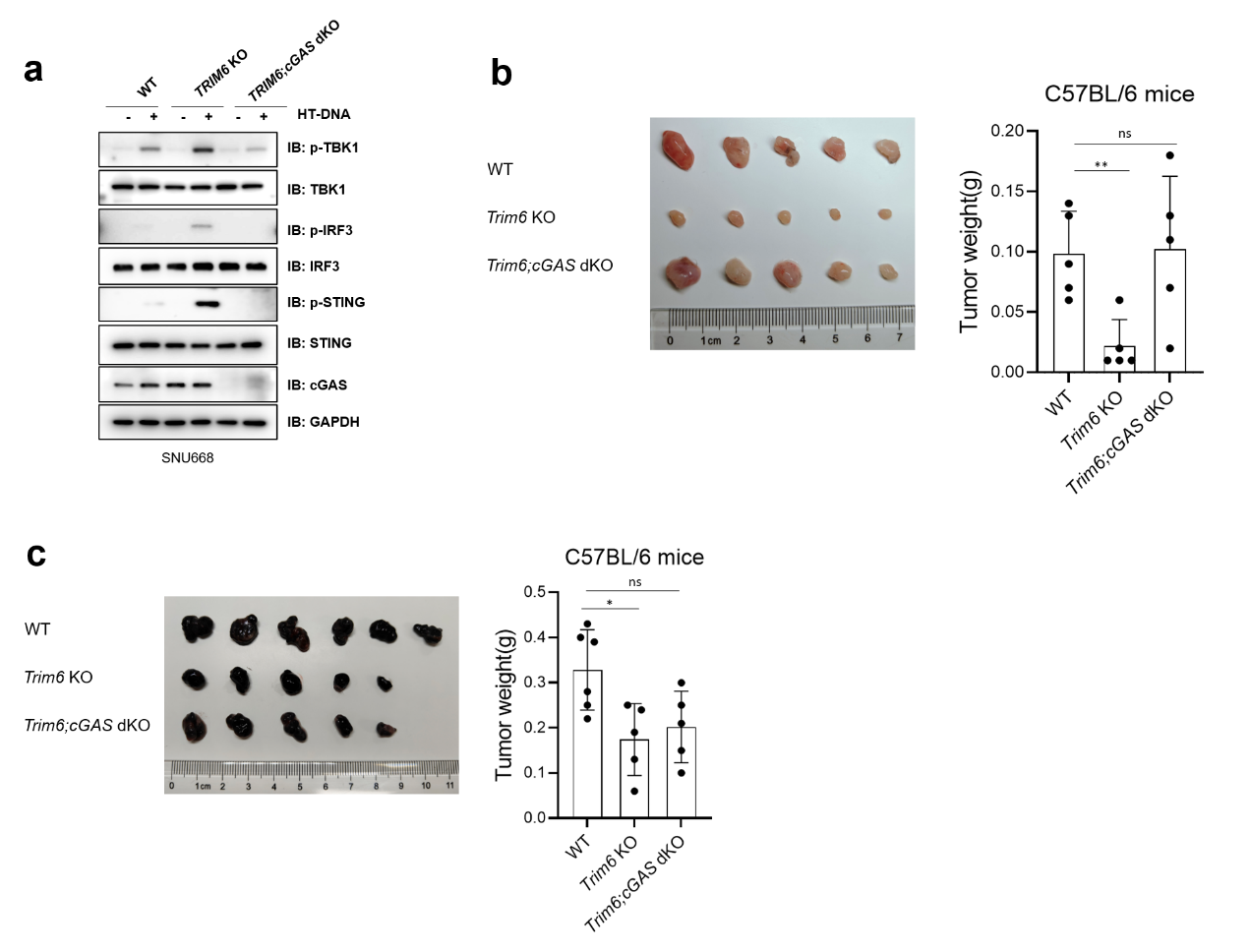


**Supplementary Figure S6. *Trim6* knockout activates cGAS-STING pathway.**

**a** IB analysis of WT, *TRIM6* KO and *cGAS* dKO SNU668 cells transfected with HT-DNA (2 µg/ml) for 6h. **b** and **c** Tumor growth and mass of WT, *Trim6* KO and *Trim6; cGAS* dKO MTC (**b**) and B16F10 cells (**c**). ns, no significance; **p* < 0.05, ***p* < 0.01, ****p* < 0.001


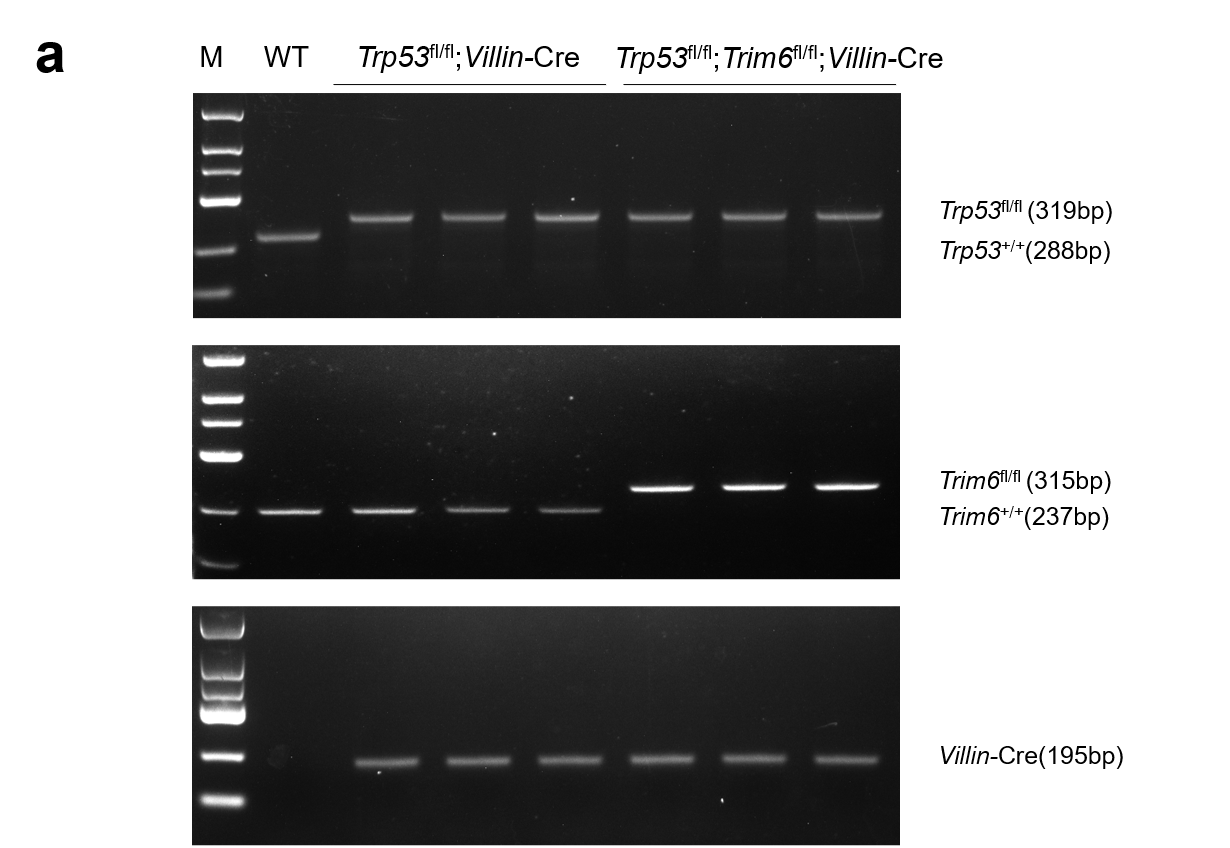


**Supplementary Figure S7. Genetic validation of *Trim6*-targeted conditional knockout mice**

**a** Agarose gel electrophoresis demonstrating allele-specific amplification of wild-type (WT, 237-bp for *Trim6*/288-bp for *Trp53*) and floxed (315-bp for *Trim6*/319-bp for *Trp53*) alleles using LoxP site-flanking primers in murine tail genomic DNA.

**Supplementary Tables**

**Supplementary Table S1. Sequence of sgRNA and shRNA primers.**

| **1. CRISPR gRNAs** | | |
| --- | --- | --- |
| *TRIM6* #1 | | CCAGATGCCGATTAGGCCGC |
| *TRIM6* #2 | | GCGAGAGCTCATCTCGGATC |
| *CGAS* #1 | | ATGATATCTCCACGGCGGCG |
| *CGAS* #2 | | CCGCCGTGGAGATATCATCG |
| Mouse *Trim6* #1 | | TAAGCAGCTCCGAAGCATCC |
| Mouse *Trim6* #2 | | GAGACTCCTGGAACATGTCC |
| Mouse *Cgas* #1 | | GTCGGGGCGCGCTTCGCGGA |
| Mouse *Cgas* #2 | | CCGAGGCGCGGAAAGTCGTA |
| **2. ShRNAs** | | |
| *TRIM6*-Sh1-S | CCGGCCGGAGACAAGTGAGGTTTCTCGAGAAACCTCACTTGTCTCCGGTTTTTG | |
| *TRIM6*-Sh1-A | AATTCAAAAACCGGAGACAAGTGAGGTTTCTCGAGAAACCTCACTTGTCTCCGG | |
| *TRIM6-*Sh2-S | CCGGGAATCCACACACAGCTAATTTCTCGAGAAATTAGCTGTGTGTGGATTCTTTTTG | |
| *TRIM6*-Sh2-A | AATTCAAAAAGAATCCACACACAGCTAATTTCTCGAGAAATTAGCTGTGTGTGGATTC | |

**Supplementary Table S2. Primers for RT-qPCR and BSP-PCR.**

| **RT-qPCR** | **Forward primers** | **Reverse primers** |
| --- | --- | --- |
| Human *TRIM6* | CGTGACCCTGAATCCACACA | AGCCCAGGACACTACAGTCA |
| Human *CGAS* | ACATGGCGGCTATCCTTCTCT | GGGTTCTGGGTACATACGTGAAA |
| Human *ISG15* | CGCAGATCACCCAGAAGATCG | TTCGTCGCATTTGTCCACCA |
| Human *IFNβ1* | AGGACAGGATGAACTTTGAC | TGATAGACATTAGCCAGGAG |
| Human *CXCL10* | GTGGCATTCAAGGAGTACCTC | TGATGGCCTTCGATTCTGGATT |
| Human *GAPDH* | GAGTCAACGGATTTGGTCGT | TTGATTTTGGAGGGATCTCG |
| Mouse *Trim6* | CGTGACCCTGAATCCACACA | AGCCCAGGACACTACAGTCA |
| Mouse *Cgas* | TTTCAAGGAGAGACCTGGCG | TTCTGCAGCATTTTGCTCCG |
| Mouse *Ifnβ1* | ATGAACTCCACCAGCAGACAG | ACCACCATCCAGGCGTAGC |
| Mouse *Ifna4* | CCTGTGTGATGCAGGAACC | TCACCTCCCAGGCACAGA |
| Mouse *Ccl5* | TCACCATATGGCTCGGACACCAC | TTGGCACACACTTGGCGGTTC |
| Mouse *Cxcl10* | ATCATCCCTGCGAGCCTATCCT | GACCTTTTTTGGCTAAACGCTTTC |
| Mouse *Isg15* | GAGCTAGAGCCTGCAGCAAT | TCACGGACACCAGGAAATCG |
| Mouse *Gapdh* | GGAGCGAGACCCCACTAACA | ATGCCAGTGAGCTTCCCGTTCAG |
| **BSP-PCR** | **Forward primers** | **Reverse primers** |
| *TRIM6* | CGTGACCCTGAATCCACACA | AGCCCAGGACACTACAGTCA |
